# The Identification of Metal Ion Ligand-Binding Residues by Adding the Reclassified Relative Solvent Accessibility

**DOI:** 10.3389/fgene.2020.00214

**Published:** 2020-03-19

**Authors:** Xiuzhen Hu, Zhenxing Feng, Xiaojin Zhang, Liu Liu, Shan Wang

**Affiliations:** College of Sciences, Inner Mongolla University of Technology, Hohhot, China

**Keywords:** metal ion ligand, binding residues, relative solvent accessibility, secondary structure, position weight matrix

## Abstract

Many proteins realize their special functions by binding with specific metal ion ligands during a cell’s life cycle. The ability to correctly identify metal ion ligand-binding residues is valuable for the human health and the design of molecular drug. Precisely identifying these residues, however, remains challenging work. We have presented an improved computational approach for predicting the binding residues of 10 metal ion ligands (Zn^2+,^ Cu^2+^, Fe^2+^, Fe^3+^, Co^2+^, Ca^2+^, Mg^2+^, Mn^2+^, Na^+^, and K^+^) by adding reclassified relative solvent accessibility (RSA). The best accuracy of fivefold cross-validation was higher than 77.9%, which was about 16% higher than the previous result on the same dataset. It was found that different reclassification of the RSA information can make different contributions to the identification of specific ligand binding residues. Our study has provided an additional understanding of the effect of the RSA on the identification of metal ion ligand binding residues.

## Introduction

Proteins act as an indispensable material in the life process. However, many special functions of protein are realized by binding with specific ligands, and more than one-third of the proteins need to bind with metal ion ligands. Thus, depending on the interaction between the metal ion ligands and specific binding residues, many metal ion ligands can affect the special protein functions (Caspers et al., 1990; Supek et al., 1997; Selvarengan and Kolandaivel, 2005). For instance, Mn^2+^ is used as catalyst in photosynthesis (Degtyarenko, 2000; Reed and Poyner, 2000), Ca^2+^ can lead to anxiety and Alzheimer’s disease (Jiang et al., 2015; Cao et al., 2017), and Cu^2+^ can cause Coronary Heart Disease (Sodhi et al., 2004; Lin et al., 2005). The basic principle of molecular drug design is that the interaction between the receptor and ligand must conform to the “Lock and Key Model,” and the interaction between the protein and ion ligands we studied also conforms to the “Lock and Key Model.” In the experiment of molecular drug design, protein crystallization, structure confirmation, and the interaction between ligands and protein residues are required. Thus, the experimental method is a time-consuming and expensive process, and it cannot be processed in batches, however, theoretical prediction of binding residues between proteins and ligands can overcome these shortcomings, and accurate prediction can provide theoretical information for drug design experiments. Therefore, correctly identifying metal ion ligand-binding residues is helpful for the human health and the design of molecular drug.

In the past two decades, experimental methods have been developed to identify metal ion ligand-binding residues, such as the Nuclear Magnetic Resonance Spectroscopy (Sletten, 1997) and fluorescence method (Kawahashi, 2003). However, due to the time-consuming nature and other limitations of experimental methods, the high-throughput computational methods were developed to predict the binding residues of metal ion ligands. Among the computational methods, many efforts were made to improve the databases, feature parameters, and algorithms. First, the databases were generally acquired from Protein Data Bank (PDB) (Tainer et al., 1991; Bernstein et al., 1997; Sodhi et al., 2004; Lin et al., 2005; Bordner, 2008; Babor et al., 2010; Lu et al., 2012), Structural Classification of Protein (SCOP) (Hubbard et al., 1997; Sodhi et al., 2004; Chauhan et al., 2010; Sobolev and Edelman, 2013), Ligand Protein Contact (LPC) (Sobolev et al., 1999; Chauhan et al., 2010), and BioLip (Yang et al., 2013a, b; Hu et al., 2016a, b, Wang et al., 2019). Second, the feature parameters generally contained the composition information of the amino acid (Cao et al., 2017; Wang et al., 2019), hydrophilicity-hydrophobicity (Lin et al., 2005; Lin et al., 2006; Cao et al., 2017), charge (Lin et al., 2005; Cao et al., 2017; Wang et al., 2019), position specific score matrix (PSSM) (Hu et al., 2016a), relative solvent accessibility (RSA) (Lin et al., 2006; Hu et al., 2016a; Cao et al., 2017; Wang et al., 2019) and three-dimensional structure information (Babor et al., 2010; Roy et al., 2012; Yang et al., 2015; Hu et al., 2016a). Finally, the classification algorithms used were artificial neural network (ANN) (Lin et al., 2005), Support Vector Machine (SVM) (Lin et al., 2006; Jiang et al., 2015; Cao et al., 2017; Hu et al., 2016a), Naïve Bayes (Ebert and Altman, 2010), COFACTOR (Lin et al., 2006; Yang et al., 2015), TargetSeq, TargetCom (Hu et al., 2016b), COACH (Yang et al., 2015), and SMO (Wang et al., 2019). Among the three aspects in the prediction mentioned above, the key step of feature extraction was generated by one of two ways: (1) the three-dimensional structure information or (2) primary sequence information of the protein. However, the precise three-dimensional structure information of many proteins was not available in the recent databases. Thus, feature extraction from sequence information is more popular in current research. Among the sequence information, RSA is one of the important parameters. In the previous works, researchers only divided it to burial and exposure by a certain threshold. However, the effects of different classifications of the RSA on prediction results have not been explored. In this paper, based on the semi-manually curated database of BioLip for biologically relevant ligand–protein interactions, we performed a statistical analysis for RSA and further reclassified the RSA. By integrating the optimized sequence information, we mainly used the Gradient Boosting Machine (GBM) algorithm and obtained better predicted results by using fivefold cross-validation and an independent test.

## Materials and Methods

### Benchmark Dataset

We selected non-redundant datasets of metal ion-binding proteins that were constructed in our group (Cao et al., 2017; Wang et al., 2019). The benchmark datasets were entirely from the BioLip database (Yang et al., 2013a). The proteins were filtered with a resolution less than 3 Å, the length of sequences was greater than 50, and the sequence identity was below 30%. Among the ∼250 ligands, there were only 10 ligands that could meet the above conditions to contribute to our further statistical analysis and prediction. The statistical information of the datasets containing ten metal ion ligands is shown in Table 1. In the protein sequence, residue binding with ion ligands was not only determined by the residue itself but also by how this was affected by the surrounding residues. Thus, a sliding window method was used to cut the protein sequence into overlapping residue segments with different sizes ranging from 5 to 21. In order to ensure that each residue was in the center of the segments, we added (L-1)/2 dummy residues “X” at both terminals of the proteins, where L was the window length. The optimal window length for each ligand was determined based on the evaluation results of the proposed computation method. If a binding residue was located at the segment center, it was defined as a positive sample; otherwise, it was defined as a negative sample. The number of non-binding segments was much larger than that of the binding segments, which led to a heavy imbalance in the datasets (Table 1). According to the methods of previous works (Yen and Lee, 2006; Roy et al., 2015), we took the number of positive samples as the standard and randomly extracted the equal number of negative samples. In this way, the negative samples were randomly selected 10 times to ensure the credibility of the results. Finally, we averaged the 10 results to calculate our overall accuracy.

**TABLE 1:** The benchmark datasets of 10 metal ion ligands.

Metal ion ligand	Number of chains	P	N	L
Zn^2+^	1428	6408	405113	7
Cu^2+^	117	485	33948	13
Fe^2+^	92	382	29345	9
Fe^3+^	217	1057	68829	9
Co^2+^	194	875	55050	11
Ca^2+^	1237	6789	396957	9
Mg^2+^	1461	5212	480307	9
Mn^2+^	459	2124	156625	7
Na^+^	78	489	27408	9
K^+^	57	535	18777	11

*P is the number of the binding segments of metal ion ligands, N is the number of the non-binding segments of metal ion ligands, and L is the optimal window length.*

### Selection and Extraction of Feature Parameters

According to the biological background of protein–ligand interactions and the statistical analysis of protein sequences, we extracted features of the position conservation information, which was acquired from the protein backbone and side chains.

#### Secondary Structure and Relative Solvent Accessibility

Analyzing the three-dimensional (3D) structure of a protein is critical to the understanding of its function. However, 3D models of only a small fraction of the sequenced proteins were made. The prediction of a secondary structure and RSA is a crucial step from the sequence to the 3D structure, reflecting the spatial structure information of the backbone and side chains, respectively. We therefore selected the predicted secondary structure information and RSA information. The prediction was helpful when simplifying the problem from the 3D structure to sequence information (Chen and Zhou, 2005; Lin et al., 2005; Hu et al., 2016a, b; Cao et al., 2017; Wang et al., 2019). In this paper, they were predicted by using ANGLOR software (Wu and Zhang, 2008). We obtained three secondary structure types, including alpha-helix (H), beta-strand (E), and coil (C). The relative solvent accessibility (RSA) was generally represented as a Boolean value, indicating whether the residue was buried (RSA < 0.25) or exposed (RSA > 0.25).

#### Physicochemical Properties of Amino Acids

Physicochemical properties affected the protein–ligand interactions, and different physicochemical properties of amino acids were caused by their different side chains (Lin et al., 2005, 2006; Cao et al., 2017; Wang et al., 2019). Metal ion ligands bind to a residue, probably preferring to bind to a specific side-group of this residue. The information from the side chains is therefore important for the prediction of metal ion ligand-binding residues. Since different standards can cause different classifications, the amino acids were divided into six categories according to the hydrophilicity and hydrophobicity (Panek and Eidhammer, 2010) (Supplementary Figure S1) and three categories according to the charge (Taylor, 1986) (Supplementary Figure S2).

#### Construction of Position Weight Matrix

The ion-binding residues tend to be more conserved than others during the process of evolution, and the residue conservation is a crucial indicator for the presence of functionally important residues. The PWSM has been successfully used in the prediction of transcription factor binding sites and ligand binding sites (Kel et al., 2003; Hu et al., 2016a). Thus, the position weight scoring matrix (PWSM) was used to extract the position conservation information of the basic feature parameters, and the scoring matrix based on amino acid residues was constructed from the sequence segments with a specific window length. The position-specific occurrence frequency of an amino acid is calculated as follows:

(1) $$P_{i\,j}=\frac{n_{i\,j}+\sqrt{N_{i}}/21}{N_{i}+\sqrt{N_{i}}}$$

where *i* is the position index in the sequence segment, *j* is one of the 20 kinds of amino acids or vacancy, *n*_*ij*_ is the frequency of the *j*th amino acids at the *i*th position, and *N*_*i*_ is total number of all amino acids occurring at the *ith* position. The position weight matrix is then calculated as follows:

(2) $$W_{i\,j}=\log\frac{P_{i\,j}}{P_{o\,j}}$$

where *P*_*oj*_ is background probability of the *jth* amino acid. Therefore, based on the positive and negative training sets, two standard scoring matrices can be obtained. In a testing set, we got 2^∗^L dimensional values for every sequence segment. Finally, the 5^∗^2L dimensional values from the above five features can be used as the input parameters in the subsequent algorithm.

### Gradient Boosting Machine

The Gradient Boosting Machine (GBM) is an improved Boosting algorithm proposed by Friedman (2001, 2002), Rawi et al. (2018) and Jain et al. (2018). The GBM algorithm is different from the original Boosting algorithm. The core of the Boosting algorithm is to set different weights to different samples during the iterative process. Based on the results of the previous iteration, the Boosting algorithm will increase the weight of wrong classification samples and reduce the weight of correct classification samples. Then, a weak classifier will be generated in each iterative process; after m iterations, a strong classifier an improved performance will be obtained by setting weight for each weak classifier. In the iterative process, GBM algorithm classifies the sample residual of the previous iteration and not the sample itself. After the end of the iteration, our classifier *F*_*m*_(*x*) was obtained as Equation (3), where *m* is the number of iterations in the calculation process, ρ_*m*_ is the weight value and also the distance of the loss function decreases in its gradient direction, and *h*_*m*_(*x*) is the function that fits the sample residuals in the iterations.

(3) $$F_{m}\,\left(x\right)=F_{m-1}\,\left(x\right)+\rho_{m}\,h_{m}\,\left(x\right)$$

In addition, the GBM algorithm can handle mixture data and its robustness against outliers in the output space is very strong. In this paper, we implemented the GBM algorithm in the R platform by using the “gbm” package. In the classifier, parameters were optimized: “n.trees” ranged from 1 to 500, “n.minobsinnode” ranged from 10 to 50, “interaction.depth” ranged from 3 to 9, and “shrinkage” ranged from 0.01 to 0.1.

### The Validation and Evaluation Metrics

As general validation methods, cross-validation and independent tests have been commonly used in previous literature (Hu et al., 2016a, b; Sun et al., 2016; Cao et al., 2017; Wang et al., 2019). In the five cross-validations, the dataset was randomly divided into five equal subsets. Four subsets were then used as training sets, and the remaining subset was used as a testing set. This process was repeated five times in such a way that each subset was used once for testing, and the average performance of the five subsets was then taken as the final performance.

**FIGURE 1:**
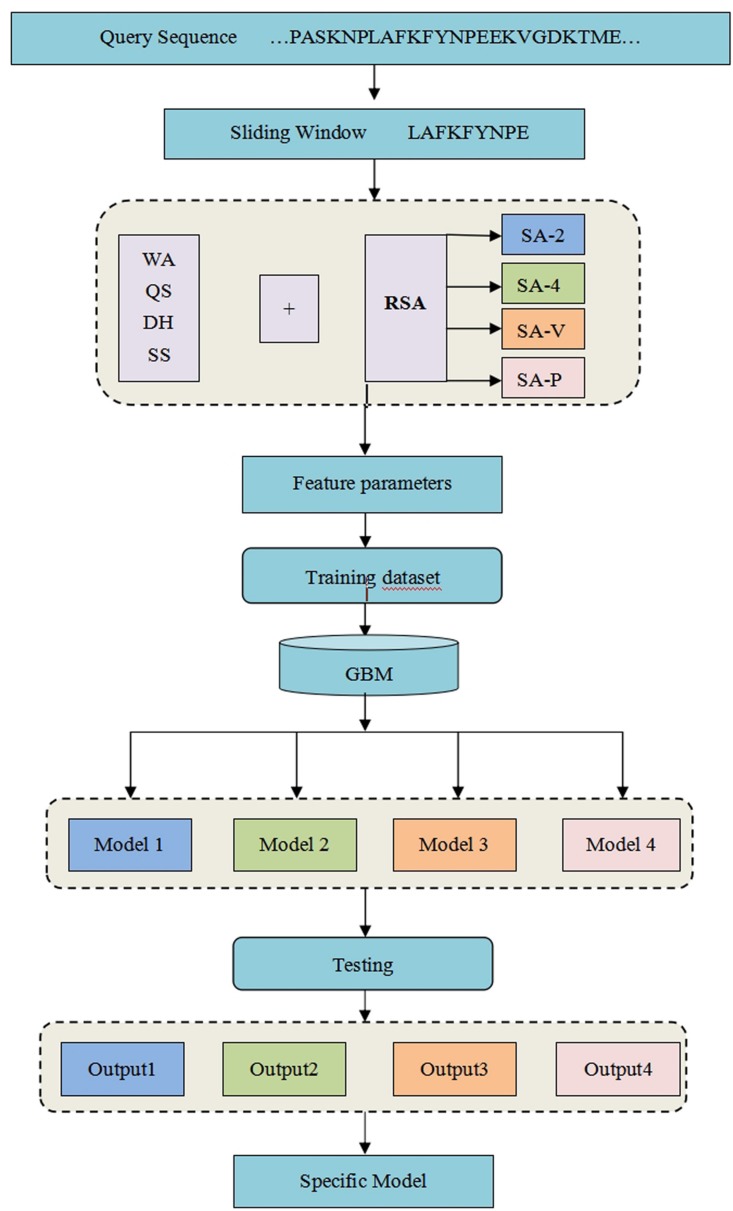
Flowchart of the method for the identification of metal ion ligand-binding residues.

We used several following metrics to evaluate our proposed method: sensitivity (Sn), specificity (Sp), False positive rate (FPR), accuracy of prediction (Acc), and Matthew’s correlation coefficient (MCC). They are defined as follows:

(4) $$S_{n}=\frac{T\,P}{T\,P+F\,N}\times 100\%$$

(5) $$S_{p}=\frac{T\,N}{T\,N+F\,P}\times 100\%$$

(6) $$F\,P\,R=\frac{F\,P}{T\,N+F\,P}\times 100\%$$

(7) $$A\,c\,c=\frac{T\,P+T\,N}{T\,P+T\,N+F\,P+F\,N}\times 100\%$$

(8) $$M\,C\,C=\frac{\left(T\,P\times T\,N\right)-\left(F\,P\times F\,N\right)}{\sqrt{\left(T\,P\times F\,P\right)\,\left(T\,P\times F\,N\right)\,\left(T\,N\times F\,P\right)\,\left(T\,N\times F\,N\right)}}$$

where TP is the number of correctly predicted metal ion ligand binding residues, FN is the number of binding residues predicted as non-binding residues, TN is the number of correctly predicted non-binding residues, and FP is the number of non-binding residues predicted as binding residues. To explain the above prediction method more directly and clearly, see our detailed flowchart in Figure 1.

## Results and Discussion

### The Classification of Relative Solvent Accessibility

For each metal ion ligand, based on the optimized window length, we gradually added the parameters from the position conservation information of amino acids (WA), hydrophilic-hydrophobic (QS), charge (DH), secondary structure (SS), and RSA to the GBM algorithm. It was found that the predicted result was significantly improved by successively adding each of the features.

#### Predicted Results for K^+^ Ligand Binding Residues

Table 2 shows the prediction results of the K + ligand by gradually adding parameters to the model. By gradually adding parameters to the model, we found that the different parameters had different effects on the predicted results. In this work, we used the initial classification of Boolean value thresholds (marked as SA_2) and added it to the model; the predicted result was significantly improved, and the Acc and MCC increased by nearly 12 and 24%, respectively. However, the predicted results did not change much by adding other parameters. It indicated that the RSA played an important role in the whole parameters for identifying the metal ion ligand-binding residues.

**TABLE 2:** Predicted results for K^+^ ligand-binding residues.

Feature parameter	Sn (%)	Sp (%)	FPR (%)	Acc (%)	MCC
WA	60.7	60.2	39.8	60.5	0.209
WA + QS	63.2	60.2	39.8	61.7	0.234
WA + QS + DH	65.4	61.9	38.1	63.6	0.273
WA + QS + DH + SS	73.8	58.5	41.5	66.2	0.327
WA + QS + DH + SS + SA_2	80.2	76.3	23.7	78.2	0.565

#### Statistical Analysis of the Relative Solvent Accessibility

**FIGURE 2:**
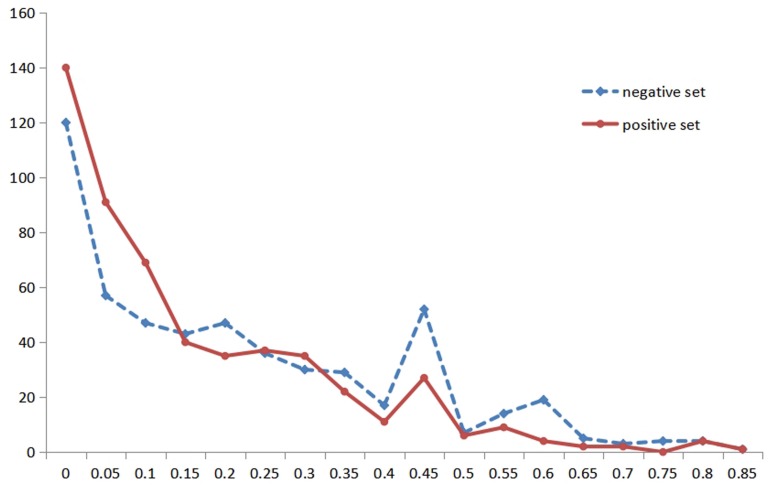
The statistical distribution of relative solvent accessibility in positive and negative set for K^+^ ligand. Note: the abscissa axis is the values of the relative solvent accessibility, and the ordinate is the number of amino acids corresponding to each predicted value. The solid red line represents the positive set, and the dotted blue line represents the negative set.

Due to the importance of RSA and the particularity of metal ion ligands, we performed the statistical analysis of the RSA information for different metal ion ligands. Then, we found that the classification was not the same for different metal ion ligands. Therefore, we reclassified the thresholds of the Boolean value for different metal ion ligands. For instance, Figure 2 shows the statistical distribution of the RSA in a positive set and negative set for the K^+^ ligand (the statistical distribution of other metal ion ligands is shown in Supplementary Material 1). In Figure 2, the abscissa indicates the predicted values of amino acid RSA; the ordinate indicates the number of amino acids corresponding to each predicted value in the positive and negative samples.

If it is reclassified by the distribution, it can be divided into four regions (marked as SA_V), namely [0, 0.15), [0.15, 0.25), [0.25, 0.35), and [0.35, 0.85], which are represented by h(x) and four letters.

$$h\,\left(x\right)=\left\{ \begin{array}{c} A,x\in \left[0,0.15\right)\\ B,x\in \left[0.15,0.25\right)\\ C,x\in \left[0.25,0.35\right)\\ D,x\in \left[0.35,0.85\right] \end{array}\right.$$

If it is reclassified according to the peak value, it can be divided into four regions (marked as SA_P), namely [0, 0.15), [0.15, 0.4), [0.4, 0.5), and [0.5, 0.85], which are expressed by y(x) and four letters.

$$y\,\left(x\right)=\left\{ \begin{array}{c} I,x\in \left[0,0.15\right)\\ K,x\in \left[0.15,0.4\right)\\ L,x\in \left[0.4,0.5\right)\\ M,x\in \left[0.5,0.85\right] \end{array}\right.$$

Besides, we also used the previous four regions (Cao et al., 2017), which were suitable for most metal ion ligands (marked as SA_4), namely [0, 0.2], (0.2, 0.45], (0.45, 0.6], and (0.6, 0.85], which were represented by g(x) and four letters. The four kinds of grouping methods (SA_2, SA_4, SA_P, and SA_V) of other metal ion ligands are shown in Supplementary Material 2.

$$g\,\left(x\right)=\left\{ \begin{array}{c} E,x\in \left[0,0.2\right]\\ F,x\in \left(0.2,0.45\right]\\ G,x\in \left(0.45,0.6\right]\\ H,x\in \left(0.6,0.85\right] \end{array}\right.$$

### The Predicted Results of Four General RSA Classifications

Then, for each metal ion ligand, four different classification groups of RSA were added to the parameters, and four general prediction models were obtained. The four different predicted results of K^+^ ligand binding residues are shown in Table 3.

**TABLE 3:** Predicted results of K^+^ ligand-binding residues.

SA classification	Sn (%)	Sp (%)	FPR (%)	Acc (%)	MCC
SA_2	80.2	76.3	23.7	78.2	0.565
SA_4	85.4	81.9	18.1	83.6	0.673
SA_V	87.5	85.0	15.0	86.3	0.725
SA_P	81.7	77.4	22.6	79.5	0.591

We found that the predicted results of the same metal ion ligand were different for the four general prediction models, and the optimal predicted results of ten metal ion ligand-binding residues were from the differently specific prediction model. An additional file shows this in more detail (see Supplementary Material 3). For example, the K^+^ ligand obtained the optimal predicted result from the specific classification namely SA_V, but the Fe^2+^ ligand obtained this from SA_4.

### The Optimal Predicted Results of Ten Metal Ion Ligand-Binding Residues

By comparing the four general prediction models, the optimal predicted results for ten metal ion ligand-binding residues were obtained and listed in Table 4.

**TABLE 4:** The optimal predicted results of 10 metal ion ligand-binding residues and corresponding specific classifications of relative solvent accessibility.

Ligand	SA classification	Sn (%)	Sp (%)	FPR (%)	Acc (%)	MCC
Zn^2+^	SA_4	92.6	90.3	9.7	91.5	0.829
Cu^2+^	SA_4	94.0	94.2	5.8	94.1	0.883
Fe^2+^	SA_4	99.2	100	0	99.6	0.992
Fe^3+^	SA_V	88.6	91.4	8.6	90.0	0.801
Co^2+^	SA_V	79.8	89.6	10.4	84.7	0.697
Ca^2+^	SA_2	76.6	79.2	20.8	77.9	0.558
Mg^2+^	SA_4	91.6	91.5	8.5	91.6	0.831
Mn^2+^	SA_P	81.3	88.3	11.7	84.8	0.698
Na^+^	SA_V	85.9	84.0	16.0	85.0	0.700
K^+^	SA_V	87.5	85.0	15.0	86.3	0.725

Based on the different classifications of RSA, we obtained the optimal predicted results of ten metal ion ligand-binding residues and corresponding specific prediction models.

### The Predicted Results (by Use of the Boruta Algorithm)

We used the 5^∗^2L dimensional features in the above calculations. However, different features made varied contributions to the predicted results, and the combination of different features did not necessarily result in a good classification performance. Therefore, we used a Boruta algorithm (Kursa and Rudnicki, 2010; Kursa et al., 2010; Feng and Li, 2017, Feng et al., 2018) to make a main feature selection. The algorithm iteratively removed the features that were less relevant than random probes. From this we could obtain the optimal features combination. The algorithm was implemented by the “Boruta” package in R environment. In this way, after a large-scale computation, the confirmed features were obtained, and the rejected features were removed from the combination of all the features. The rejected features are shown in Table 5.

**TABLE 5:** The features rejected by using the Boruta feature selection algorithm.

Metal ion ligand	Rejected features
Zn^2+^	WA6, DH4, DH12, DH13, DH14
Cu^2+^	WA2, WA5, WA6, WA8, WA15, WA18, WA19, WA20, WA21, WA22, WA23, WA24, QS1, QS2, QS3, QS4, QS5, QS6, QS7, QS8, QS9, QS10, QS11, QS15, QS16, QS17, QS18, QS19, QS20, QS22, QS23, QS24, QS25, DH1, DH2, DH3, DH4, DH5, DH6, DH7, DH8, DH9, DH10, DH11, DH12, DH15, DH16, DH17, DH18, DH19, DH20, DH21, DH22, DH23, DH24, DH25, DH26, SS1, SS2, SS3, SS4, SS5, SS6, SS8, SS13, SS14, SS15, SS16, SS17, SS21, SS22, SS26, SA1, SA2, SA3, SA4, SA5, SA6, SA7, SA8, SA9, SA10, SA12, SA21, SA23, SA24, SA25, SA26
Fe^2+^	WA1, WA2, WA4, WA8, WA12, WA13, WA14, WA15, WA17, QS1, QS2, QS3, QS4, QS5, QS6, QS7, QS8, QS9, QS10, QS11, QS13, QS14, QS17, QS18, DH1, DH2, DH3, DH4, DH5, DH6, DH7, DH8, DH11, DH12, DH13, DH14, DH15, DH16, SS2, SS4, SS9, SS10, SS11, SS12, SS13, SS15, SS16, SS17, SS18, SA 11, SA12, SA18
Fe^3+^	WA1, WA2, WA5, WA8, WA11, WA12, WA13, WA14, WA15, WA17, WA18, QS1, QS2, QS5, QS7, QS8, QS11, QS12, QS13, QS14, QS16, QS17, QS18, DH1, DH2, DH5, DH6, DH11, DH13, DH14, DH15, DH16, SA 18
Co^2+^	WA1, WA2, WA4, WA6, WA10, WA13, WA14, WA15, WA16, WA17, WA18, WA19, WA20, WA21, WA22, QS1, QS2, QS3, QS4, QS7, QS8, QS9, QS13, QS14, QS15, QS16, QS17, QS18, QS19, QS20, QS21, QS22, DH1, DH2, DH3, DH4, DH5, DH6, DH7, DH8, DH9, DH10, DH13, DH15, DH16, DH17, DH18, DH19, DH20, DH21, DH22, SS19, SS20, SS21, SA1, SA2, SA16, SA19, SA20, SA21, SA22
Mn^2+^	WA10, WA12, WA13, QS2, QS4, QS9, QS10, QS11, QS12, DH9, DH11, DH12, DH14
Na^+^	WA3, WA4, WA5, WA6, WA7, WA8, WA10, QS2, QS3, QS4, QS5, QS6, QS7, QS8, QS10, QS12, QS13, QS15, QS16, QS17, QS18, DH1, DH2, DH3, DH4, DH5, DH6, DH7, DH8, DH11, DH12, DH13, DH14, DH15, DH16, DH17, DH18, SS1, SS3, SS5, SS8, SS9, SS10, SS16, SS17, SS18, SA1, SA2, SA3, SA4, SA5, SA6, SA13, SA18
K^+^	WA1, WA2, WA3, WA5, WA6, WA7, WA8, WA9, WA10, WA13, WA14, WA18, WA19, WA21, WA22, QS1, QS2, QS3, QS4, QS5, QS6, QS7, QS8, QS9, QS10, QS13, QS14, QS15, QS17, QS18, QS19, QS20, QS21, QS22, DH1, DH2, DH3, DH4, DH5, DH6, DH7, DH8, DH9, DH13, DH14, DH15, DH16, DH17, DH18, DH19, DH20, DH21, DH22, SS1, SS2, SS3, SS4, SS5, SS6, SS21, SS22, SA1, SA2, SA4, SA5, SA6, SA8, SA10, SA13, SA14, SA17, SA18, SA19, SA20, SA21, SA22

*When “i” is an odd number, WAi, DHi, QSi, SSi, and SAi indicate the matrix value of amino acid, charge, hydrophilic-hydrophobic, secondary structure, and relative solvent accessibility at the ((i + 1)/2)th position calculated from the positive training set. When “i” is an even number, WAi, DHi, QSi, SSi, and SAi indicates the corresponding values at the (i/2)th position calculated from the negative training set.*

**FIGURE 3:**
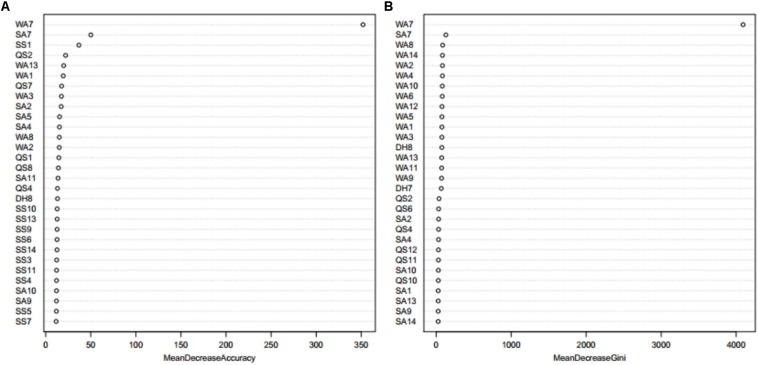
The feature importance of Zn^2+^ ligand indicated by MeanDecreaseAccuracy value **(A)** and MeanDecreaseGini value **(B)** from Random Forest. Note: the larger the MeanDecreaseAccuracy and MeanDecreaseGini values, the higher the importance of the feature parameters. WA1-WA18 is the features of amino acid, QS1-QS18 is the features of hydrophobic, DH1-DH18 is the features of charge, SS1-SS18 is the features of secondary structure, and SA1-SA18 is the features of relative solvent accessibility.

When using the Boruta algorithm to reduce the dimension of the features, it was found that the reduced dimensions of different metal ion ligands were different. For example, the dimensions of the Ca^2+^ and Mg^2+^ ligands were not reduced, the dimension of the Zn^2+^ ligand was reduced by 5 dimensions, the dimension of the Mn^2+^ ligand was reduced by 13 dimensions, etc. In order to prove the justifiability of the features eliminated by the Boruta algorithm, we analyzed the importance of the features by using the “randomForest” package in R environment. The larger the MeanDecreaseAccuracy and MeanDecreaseGini values, the higher the importance of the feature parameters. Taking the Zn^2+^ ligand as an example, it can be seen from Figure 3 that the important features of the first 30 dimensions were consistent with the confirmed features by the Boruta algorithm.

The obtained subset features were then input into the GBM, and the predicted results were shown in Table 6. Table 6 shows that we obtained similar results based on subset features. This suggested that, under the premise of ensuring the accuracy, the Boruta algorithm was efficient in its ability to reduce the dimensions of features for predicting metal ion ligand-binding residues. The decline of the subset predicted results showed that all the selected features had certain contributions to the recognition of the binding residues. In addition, the predicted results of the subset were still higher than those of SVM. Our method was therefore relatively reliable for predicting the metal ion ligand binding residues.

**TABLE 6:** Comparison of predicted results based on the full feature and Boruta’s feature.

Ligand	Feature selection	Feature dimension	Sn (%)	Sp (%)	Acc (%)	MCC
Zn^2+^	Full	70	92.6	90.3	91.5	0.829
	Boruta	65	92.7	89.1	90.9	0.818
Cu^2+^	Full	130	94.0	94.2	94.1	0.883
	Boruta	42	93.4	93.8	93.6	0.872
Fe^2+^	Full	90	99.2	100	99.6	0.992
	Boruta	40	96.1	96.1	96.1	0.921
Fe^3+^	Full	90	88.6	91.4	90.0	0.801
	Boruta	57	88.0	90.7	89.4	0.787
Co^2+^	Full	110	79.8	89.6	84.7	0.697
	Boruta	49	79.5	89.1	84.3	0.690
Ca^2+^	Full	90	76.6	79.2	77.9	0.558
	Boruta	90	76.6	79.2	77.9	0.558
Mg^2+^	Full	90	91.6	91.5	91.6	0.831
	Boruta	90	91.6	91.5	91.6	0.831
Mn^2+^	Full	70	81.3	88.3	84.8	0.698
	Boruta	57	81.4	88.0	84.7	0.695
Na^+^	Full	90	85.9	84.0	85.0	0.700
	Boruta	36	83.6	82.4	83.0	0.661
K^+^	Full	110	87.5	85.0	86.3	0.725
	Boruta	34	83.7	82.2	83.0	0.660

### The Predicted Results of GBM by Using an Independent Test

We used equal samples of positive and negative in the previous calculations. However, the positive and negative samples were not equal when we intercepted segments by using the sliding window method. In order to verify the practicability of the proposed method, we divided the total dataset into two parts: the training dataset was used to construct the predicted methods by fivefold cross-validation, and the independent testing dataset was used to test the extrapolation ability of the predicted methods. The protein chains in the independent testing dataset accounted for 20% of the total dataset, which was consistent with the published work (Cao et al., 2017). The statistical information of the datasets is shown in Table 7.

**TABLE 7:** The statistics of the training dataset and the independent testing dataset.

Ligand	Training dataset			Independent testing dataset		
	Chains	P	N	Chains	P	N
Zn^2+^	1142	5145	321,161	286	1263	83,952
Cu^2+^	93	377	27,548	24	108	6400
Fe^2+^	73	301	23,824	19	81	5521
Fe^3+^	173	859	54,945	44	198	13,884
Co^2+^	155	707	44300	39	168	10,750
Ca^2+^	989	5256	312,876	248	1533	84,081
Mg^2+^	1168	4069	384,365	293	1143	95,942
Mn^2+^	367	1685	124,543	92	439	32,082
Na^+^	62	408	22,411	16	81	4997
K^+^	45	410	14,882	12	125	3895

In the independent test, the 5^∗^2L dimension position information was input into the GBM algorithm to obtain the predicted ligand-specific models, and the testing dataset was input into the predicted model to test. The number of positive and negative samples was not balanced, and the MCC values in Table 8 therefore reflect the stability of the predicted model. In order to compare these results more obviously, we added them to Table 8. The comparative results indicated that the selected features and algorithm had better identification abilities for predicting metal ion ligand-binding residues.

**TABLE 8:** Comparison of our independent test results with previous results.

Ligand	L	Method	Sn (%)	Sp (%)	Acc (%)	MCC
Zn^2+^	7	This work	78.1	82.7	82.7	0.1865
	7	Cao et al.	94.1	84.3	**84.4**	**0.2525**
Cu^2+^	13	This work	74.1	76.8	76.7	0.1519
	13	Cao et al.	91.7	82.9	**83.0**	**0.2458**
Fe^2+^	9	This work	96.3	91.8	**91.9**	**0.3593**
	9	Cao et al.	90.1	73.6	73.9	0.1708
Fe^3+^	9	This work	90.9	83.5	**83.6**	**0.2301**
	9	Cao et al.	87.9	72.7	72.9	0.1584
Co^2+^	11	This work	76.8	83.6	**83.4**	**0.1960**
	11	Cao et al.	73.2	82.3	82.2	0.1760
Ca^2+^	9	This work	60.0	79.3	**79.0**	**0.1272**
	9	Cao et al.	59.5	79.2	78.9	0.1251
Mg^2+^	9	This work	75.7	84.0	**83.9**	**0.1724**
	9	Cao et al.	50.2	81.9	81.6	0.0871
Mn^2+^	7	This work	76.8	80.2	**80.1**	**0.1624**
	7	Cao et al.	76.5	79.8	79.8	0.1599
Na^+^	9	This work	43.2	84.5	**83.9**	**0.0947**
	9	Cao et al.	33.3	78.2	77.5	0.0348
K^+^	11	This work	51.2	73.1	**72.4**	**0.0941**
	11	Cao et al.	45.6	62.8	62.3	0.0301

*The bold values represent the best Acc and MCC values.*

### Comparison With Other Methods

**FIGURE 4:**
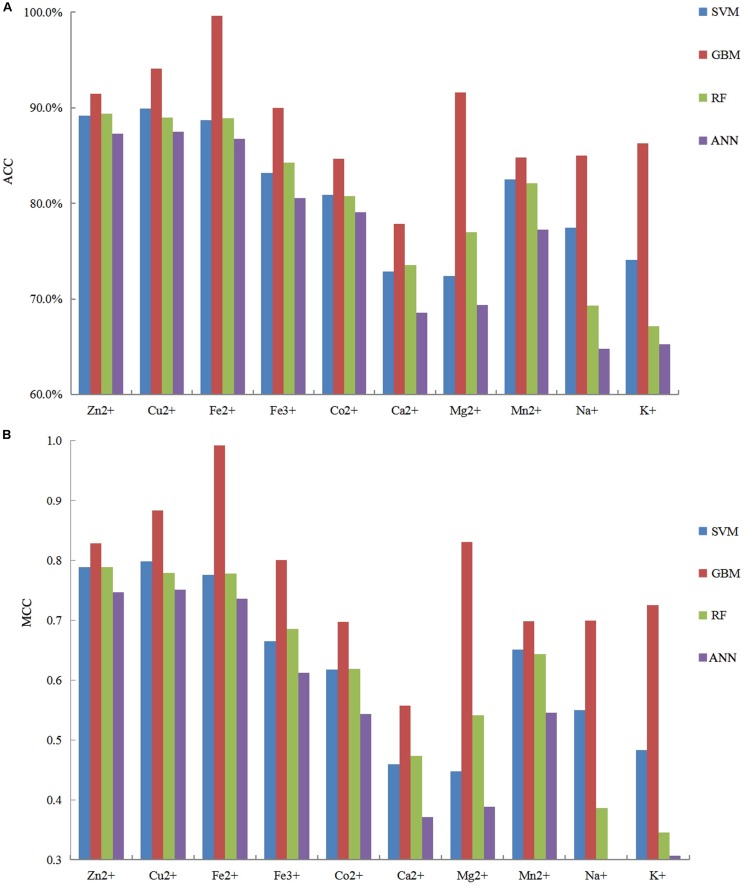
The comparison of prediction performances between several machine learning methods based on the same features by using five fold cross-validation test.

It is necessary to compare our proposed methods with previous models using the same dataset, classification strategy, and evaluation methods. For the purposes of comparison with the previous results (Cao et al., 2017; Wang et al., 2019), our predicted results of fivefold cross-validation and independent test are displayed in Tables 8, 9, respectively. Comparing the previous results in our group (Cao et al., 2017; Wang et al., 2019), most of the metal ion ligands were improved to different degrees. With the same dataset, the same feature parameters, classification strategy, and evaluation methods, we further made a comparison between the GBM algorithm and several other machine learning methods, including SVM, Random Forest, and Artificial Neural Network. Using the same features, the ACC and MCC values of each classifier for ten ligands are displayed in Figure 4. The results showed that accuracies of the GBM classifier were higher than other machine learning methods, indicating that the GBM classifier was a powerful tool for predicting metal ion ligand binding residues.

**TABLE 9:** Comparison of our optimal predicted results in fivefold cross-validation with previous results.

Ligand	Method	Sn (%)	Sp (%)	Acc (%)	MCC
Zn^2+^	This work	92.6	90.3	91.5	0.829
	Wang et al.	94.2	84.2	89.2	0.789
	Cao et al.	99.8	99.5	**99.7**	**0.993**
Cu^2+^	This work	94.0	94.2	94.1	0.883
	Wang et al.	91.3	86.8	89.0	0.782
	Cao et al.	95.5	97.1	**96.3**	**0.926**
Fe^2+^	This work	99.2	100	**99.6**	**0.992**
	Wang et al.	90.1	81.9	86.0	0.722
	Cao et al.	91.9	90.7	91.3	0.826
Fe^3+^	This work	88.6	91.4	**90.0**	**0.801**
	Wang et al.	86.2	85.5	85.9	0.717
	Cao et al.	86.9	88.7	87.8	0.756
Co^2+^	This work	79.8	89.6	**84.7**	**0.697**
	Wang et al.	75.3	86.4	80.9	0.621
	Cao et al.	80.8	85.1	83.0	0.660
Ca^2+^	This work	76.6	79.2	**77.9**	**0.558**
	Wang et al.	68.8	75.3	72.1	0.443
	Cao et al.	71.3	79.1	74.8	0.502
Mg^2+^	This work	91.6	91.5	**91.6**	**0.831**
	Wang et al.	71.1	73.1	72.1	0.442
	Cao et al.	76.6	73.9	75.3	0.505
Mn^2+^	This work	81.3	88.3	**84.8**	**0.698**
	Wang et al.	82.0	83.9	83.0	0.659
	Cao et al.	82.1	84.4	83.2	0.664
Na^+^	This work	85.9	84.0	**85.0**	**0.700**
	Wang et al.	68.9	74.0	71.0	0.430
	Cao et al.	82.2	76.2	79.4	0.586
K^+^	This work	87.5	85.0	**86.3**	**0.725**
	Wang et al.	71.6	64.5	68.0	0.362
	Cao et al.	77.3	83.2	80.3	0.607

*The bold values represent the best Acc and MCC values.*

## Conclusion

The interactions between metal ion ligands (e.g., Na^+^, Mn^2+^, Ca^2+^, K^+^, and Cu^2+^) and proteins perform key biological functions in many important life processes. Research into these metal ion ligands and functions is of significant biological import. In particular, the prediction of ligand binding residues is of great significance to the understanding of the biological functions of proteins and drug design. In this work, we predicted the binding residues of 10 metal ion ligands in the BioLip database, and we obtained improved results. According to the biological background of proteins, we selected hydrophobic polarized charges, predicted secondary structures, and RSA information as the basic information. From the statistical analysis of RSA information, we found that the reclassified RSA information has important effects on recognition of metal ion ligand-binding residues. Therefore, on the basis of primary sequence information, we extracted the important features of RSA by reclassifying the RSA as four different classifications (i.e., SA_2, SA_V, SA_P, and SA_4). Using the GBM algorithm and an overall classification strategy, we further improved the prediction success rate of metal ion ligand binding residues in the cross-validation and independent test. In the best performance, MCC values were higher than 0.558, the FPR values were lower than 20.8%, and the Acc values were higher than 77.9%. In comparison with previous results (Cao et al., 2017), our best accuracy of fivefold cross-validation was about 16% higher on the same dataset. In this research, we identified the specific contributions of different reclassified RSA to the identification of 10 ligand-binding residues. However, for the prediction performances of different ligands, there are different improvements that can indicate the differences in the ligand-binding residues. Our next step is to prove this specialty. To make our models available for other researchers, we provide our database in Supplementary Material 4 and full feature parameters in the additional material. In our future work, we will make efforts to provide a web server for the analysis method presented in this paper, which can be manipulated by readers according to their need.

## Data Availability Statement

The raw data supporting the conclusions of this article will be made available by the authors, without undue reservation, to any qualified researcher.

## Author Contributions

XH and ZF designed the experiments. XZ and XH performed the experiments. ZF improved the English. LL and SW organized the data. All authors read and approved the final manuscript.

## Conflict of Interest

The authors declare that the research was conducted in the absence of any commercial or financial relationships that could be construed as a potential conflict of interest.

## Supplementary Material

SUPPLEMENTARY MATERIAL 1 Relevant statistical analysis of physicochemical properties.

SUPPLEMENTARY MATERIAL 2 The four grouping methods of relative solvent accessibility of metal ion ligands.

SUPPLEMENTARY MATERIAL 3 Predicted results of 10 metal ion ligand-binding residues based on different classification.

SUPPLEMENTARY MATERIAL 4 The PDB IDs of protein chains in the datasets.
