# Supplementary material for: The Identification of Metal Ion Ligand-Binding Residues by Adding the Reclassified Relative Solvent Accessibility
Source: Front Genet. 2020 Mar 19;11:214. doi: 10.3389/fgene.2020.00214 (PMC7096583; doi:10.3389/fgene.2020.00214)
Supplement: SUPPLEMENTARY MATERIAL 1 — Relevant statistical analysis of physicochemical properties. [file Data_Sheet_1.doc]

**Supplementary Figures**

| **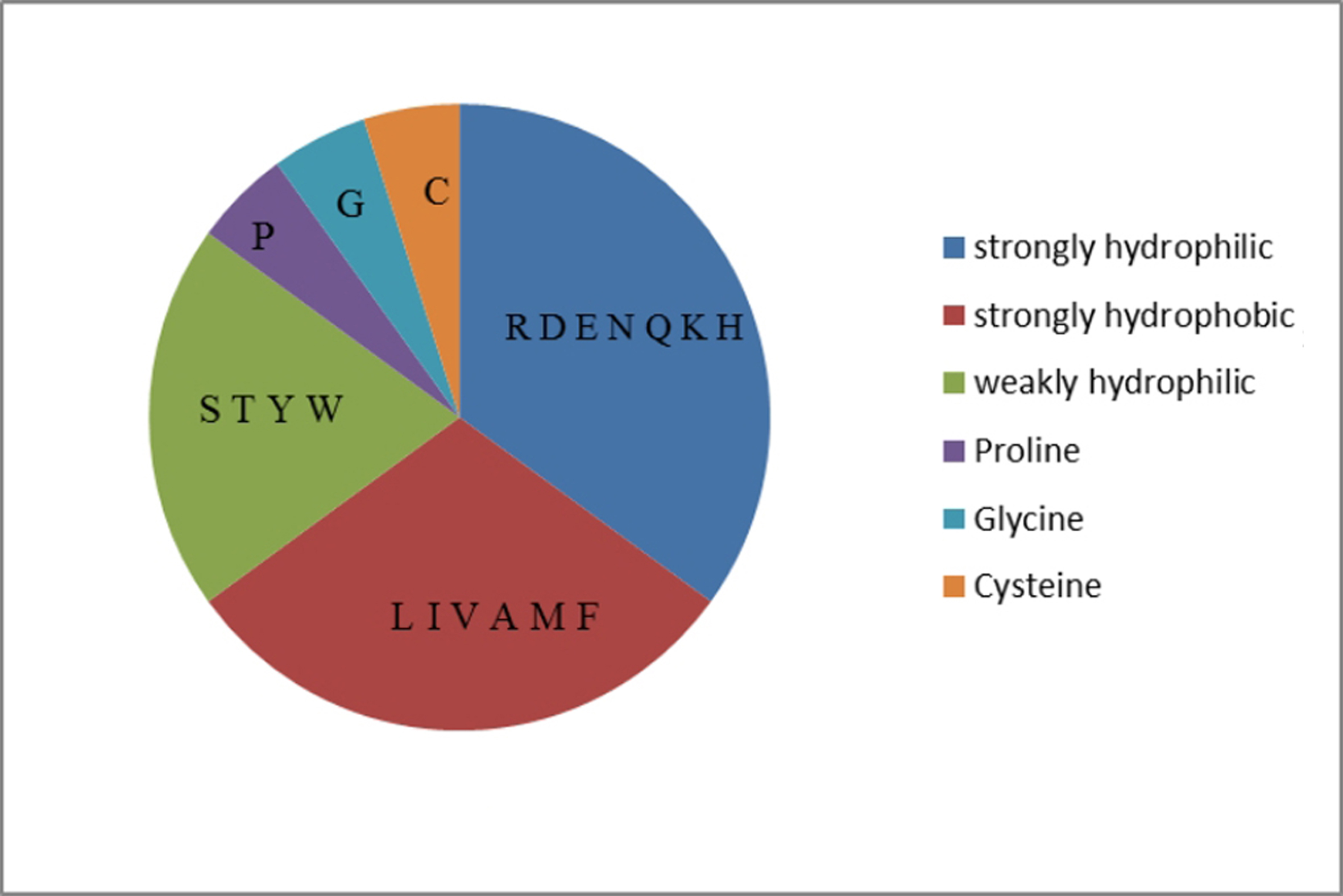** | 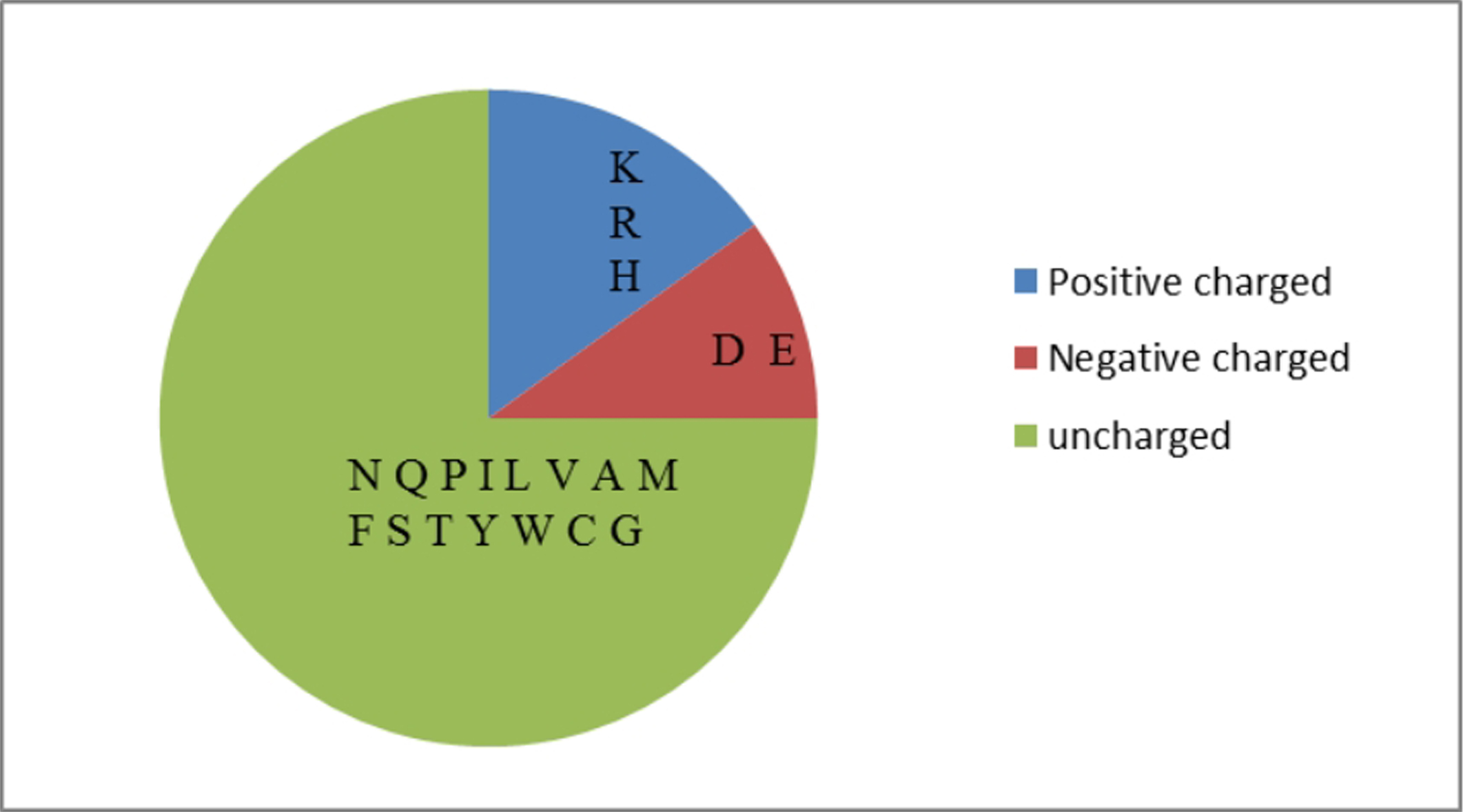 |
| --- | --- |
| **Fig.1** Hydrophilic-hydrophobic classification of amino acids | **Fig.2** The charge property of amino acids |

| 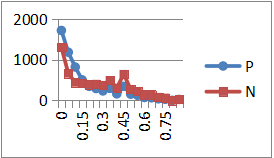 | 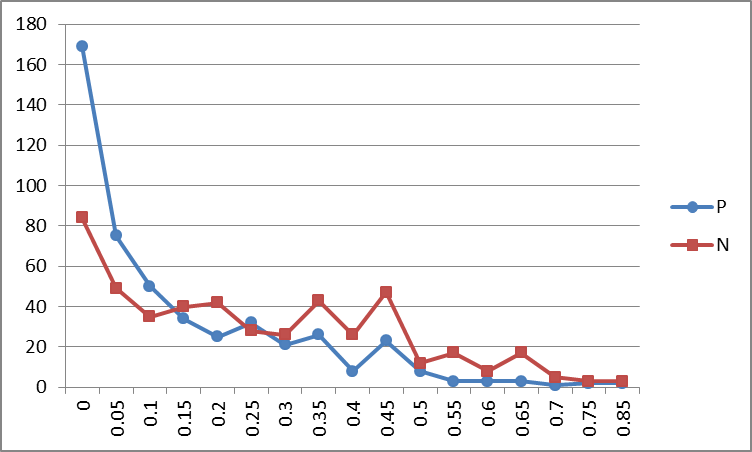 |
| --- | --- |
| **Fig.3a** The statistical distribution of relative solvent accessibility in positive and negative set for Zn^2+^ ligand | **Fig.3b** The statistical distribution of relative solvent accessibility in positive and negative set for Cu^2+^ ligand |

| 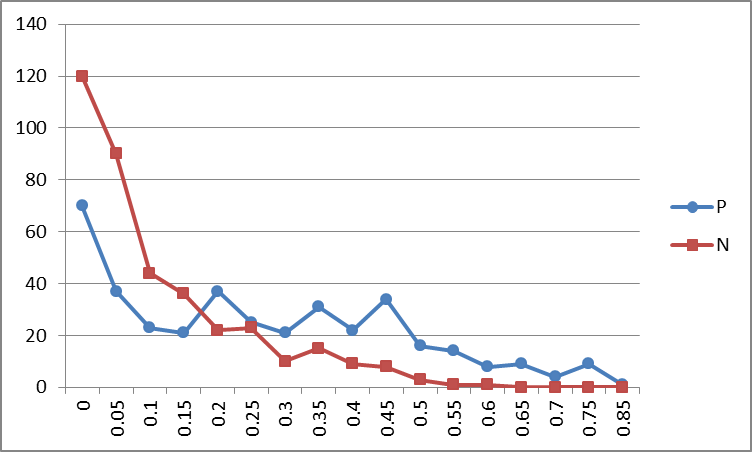 | 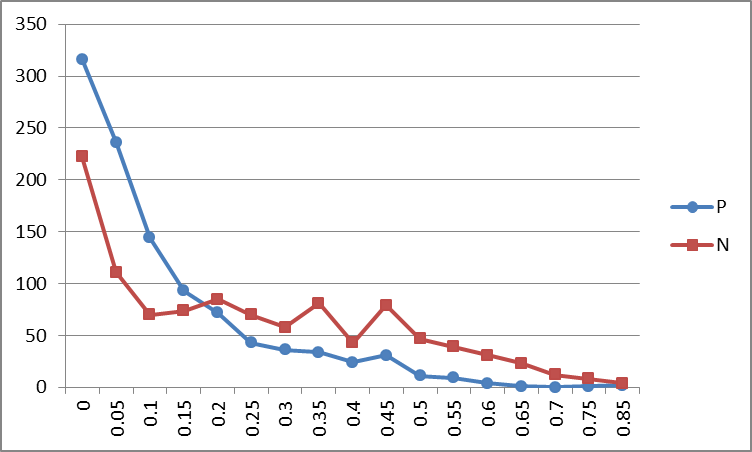 |
| --- | --- |
| **Fig.3c** The statistical distribution of relative solvent accessibility in positive and negative set for Fe^2+^ ligand | **Fig.3d** The statistical distribution of relative solvent accessibility in positive and negative set for Fe^3+^ ligand |

| 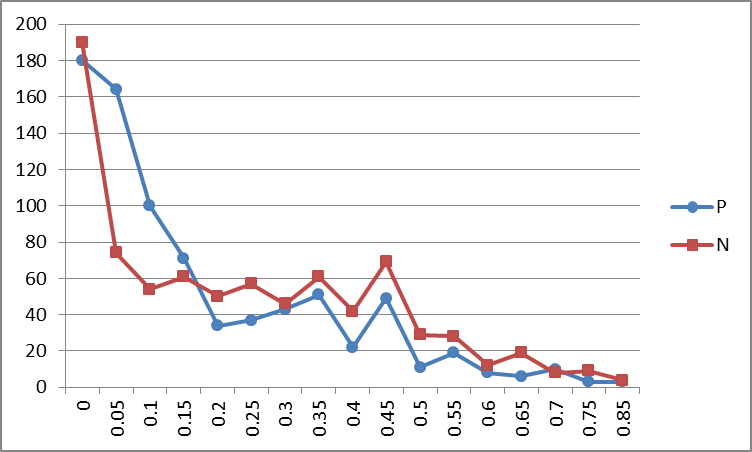 | 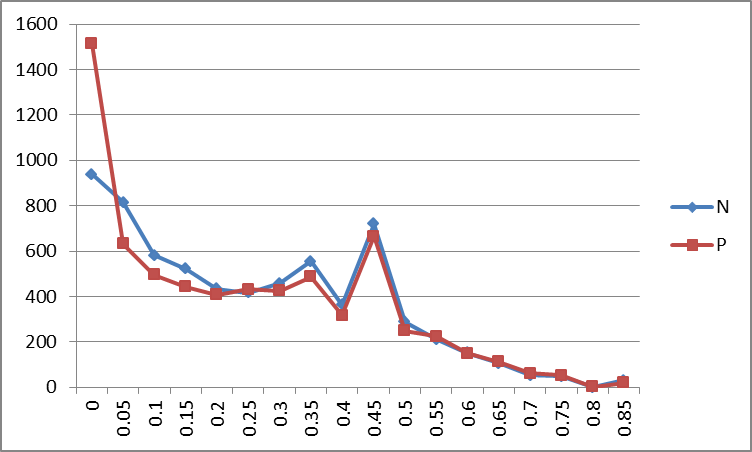 |
| --- | --- |
| **Fig.3e** The statistical distribution of relative solvent accessibility in positive and negative set for Co^2+^ ligand | **Fig.3f** The statistical distribution of relative solvent accessibility in positive and negative set for Ca^2+^ ligand |

| 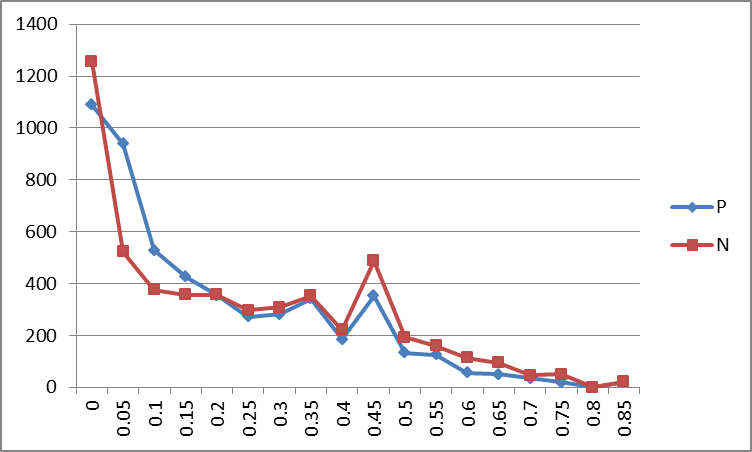 | 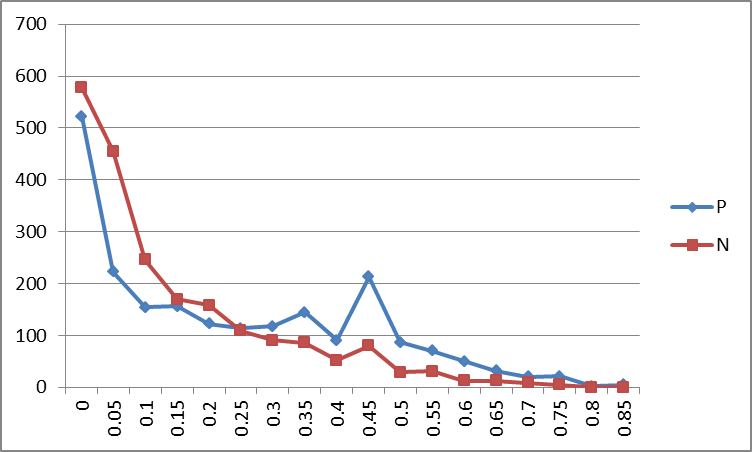 |
| --- | --- |
| **Fig.3g** The statistical distribution of relative solvent accessibility in positive and negative set for Mg^2+^ ligand | **Fig.3h** The statistical distribution of relative solvent accessibility in positive and negative set for Mn^2+^ ligand |

.

| 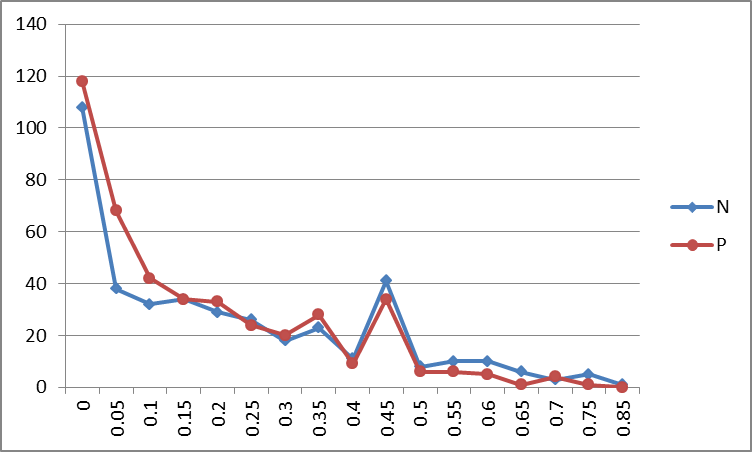 | 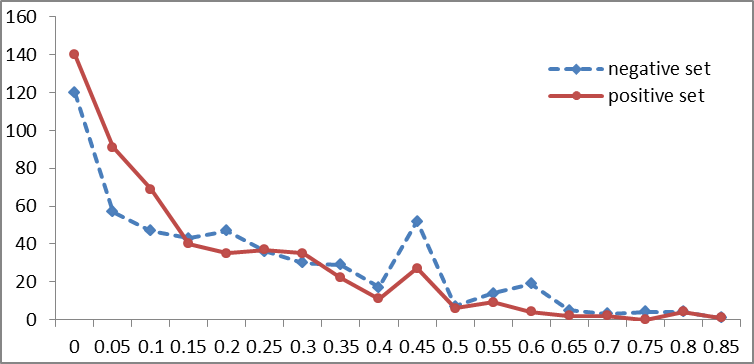 |
| --- | --- |
| **Fig.3i** The statistical distribution of relative solvent accessibility in positive and negative set for Na^+^ ligand | **Fig.3j** The statistical distribution of relative solvent accessibility in positive and negative set for K^+^ ligand |
