# Supplementary material for: The Identification of Metal Ion Ligand-Binding Residues by Adding the Reclassified Relative Solvent Accessibility
Source: Front Genet. 2020 Mar 19;11:214. doi: 10.3389/fgene.2020.00214 (PMC7096583; doi:10.3389/fgene.2020.00214)
Supplement: SUPPLEMENTARY MATERIAL 2 — The four grouping methods of relative solvent accessibility of metal ion ligands. [file Data_Sheet_2.doc]

Supplementary Tables

**Table S1 Predicted results of ten metal ion ligand binding residues based on SA_2**

| Ligand | Sn (%) | Sp (%) | FPR (%) | Acc (%) | MCC |
| --- | --- | --- | --- | --- | --- |
| Zn^2+^ | 91.5 | 90.2 | 9.8 | 90.9 | 0.817 |
| Cu^2+^ | 94.0 | 93.8 | 6.2 | 93.9 | 0.878 |
| Fe^2+^ | 96.3 | 97.4 | 2.6 | 96.9 | 0.937 |
| Fe^3+^ | 88.6 | 91.4 | 8.6 | 90.0 | 0.801 |
| Co^2+^ | 75.9 | 86.9 | 13.1 | 81.4 | 0.631 |
| Ca^2+^ | 76.6 | 79.2 | 20.8 | 77.9 | 0.558 |
| Mg^2+^ | 91.3 | 90.9 | 9.1 | 91.1 | 0.822 |
| Mn^2+^ | 82.3 | 87.4 | 12.6 | 84.8 | 0.697 |
| Na^+^ | 83.8 | 83.0 | 17.0 | 83.4 | 0.669 |
| K^+^ | 80.2 | 76.3 | 23.7 | 78.2 | 0.565 |

**Table S2 Predicted results of ten metal ion ligand binding residues based on SA_4**

| Ligand | Sn (%) | Sp (%) | FPR (%) | Acc (%) | MCC |
| --- | --- | --- | --- | --- | --- |
| Zn^2+^ | 92.6 | 90.3 | 9.7 | 91.5 | 0.829 |
| Cu^2+^ | 94.0 | 94.2 | 5.8 | 94.1 | 0.883 |
| Fe^2+^ | 99.2 | 100 | 0 | 99.6 | 0.992 |
| Fe^3+^ | 88.4 | 90.8 | 9.2 | 89.6 | 0.792 |
| Co^2+^ | 79.5 | 88.8 | 11.2 | 84.2 | 0.686 |
| Ca^2+^ | 74.3 | 78.2 | 21.8 | 76.2 | 0.525 |
| Mg^2+^ | 91.6 | 91.5 | 8.5 | 91.6 | 0.831 |
| Mn^2+^ | 81.4 | 87.9 | 12.1 | 84.7 | 0.695 |
| Na^+^ | 80.4 | 81.4 | 18.6 | 80.9 | 0.618 |
| K^+^ | 85.4 | 81.9 | 18.1 | 83.6 | 0.673 |

**Table S3 Predicted results of ten metal ion ligand binding residues based on SA_V**

| Ligand | Sn (%) | Sp (%) | FPR (%) | Acc (%) | MCC |
| --- | --- | --- | --- | --- | --- |
| Zn^2+^ | 91.5 | 90.2 | 9.8 | 90.9 | 0.817 |
| Cu^2+^ | 92.4 | 93.6 | 6.4 | 93.0 | 0.860 |
| Fe^2+^ | 96.9 | 97.4 | 2.6 | 97.1 | 0.942 |
| Fe^3+^ | 88.6 | 91.4 | 8.6 | 90.0 | 0.801 |
| Co^2+^ | 79.8 | 89.6 | 10.4 | 84.7 | 0.697 |
| Ca^2+^ | 73.1 | 77.5 | 22.5 | 75.3 | 0.506 |
| Mg^2+^ | 91.3 | 90.9 | 9.1 | 91.1 | 0.822 |
| Mn^2+^ | 82.3 | 87.4 | 12.6 | 84.8 | 0.697 |
| Na^+^ | 85.9 | 84.0 | 16.0 | 85.0 | 0.700 |
| K^+^ | 87.5 | 85.0 | 15.0 | 86.3 | 0.725 |

**Table S4 Predicted results of ten metal ion ligand binding residues based on SA_P**

| Ligand | Sn (%) | Sp (%) | FPR (%) | Acc (%) | MCC |
| --- | --- | --- | --- | --- | --- |
| Zn^2+^ | 92.8 | 88.3 | 11.7 | 90.6 | 0.812 |
| Cu^2+^ | 92.4 | 93.8 | 6.2 | 93.1 | 0.862 |
| Fe^2+^ | 96.3 | 97.1 | 2.9 | 96.7 | 0.935 |
| Fe^3+^ | 86.7 | 89.6 | 10.4 | 88.1 | 0.763 |
| Co^2+^ | 76.8 | 87.4 | 12.6 | 82.1 | 0.646 |
| Ca^2+^ | 75.0 | 78.4 | 21.6 | 76.7 | 0.535 |
| Mg^2+^ | 87.4 | 86.5 | 13.5 | 86.9 | 0.739 |
| Mn^2+^ | 81.3 | 88.3 | 11.7 | 84.8 | 0.698 |
| Na^+^ | 82.8 | 80.8 | 19.2 | 81.8 | 0.636 |
| K^+^ | 81.7 | 77.4 | 22.6 | 79.5 | 0.591 |
