# Supplementary material for: The Identification of Metal Ion Ligand-Binding Residues by Adding the Reclassified Relative Solvent Accessibility
Source: Front Genet. 2020 Mar 19;11:214. doi: 10.3389/fgene.2020.00214 (PMC7096583; doi:10.3389/fgene.2020.00214)
Supplement: SUPPLEMENTARY MATERIAL 3 — Predicted results of 10 metal ion ligand-binding residues based on different classification. [file Data_Sheet_3.doc]

The PDB IDs of protein chains in the dataset

1. Ca^2+^

5-fold cross validation set

1g9uA 1ayoB 1dctA 2c4dA 1mpxC 2wyfD 4hroB 1upsA 2c5uB 3l8yA 4q51A 3h6gB 2c3oA 3p85A 4fsoA 2pqxA 4hxbL 2clpC 3flrA 4m6xA 2jg9D 3qm2B 3htlX 3cu2B 4jjjA 1ul3B 3ge4I 3bcfA 1kkmB 1rtp2 1ys6B 1ydnC 3wd9B 4h2iA 2fkcA 3w7uA 2dbxB 1g8hB 2ejnA 4abtB 4g1eA 2ozbE 3ovsA 2bs9E 1ti2A 4fjiA 3ai7G 2gftB 1xxmD 3ayfA 4ln6G 4linF 4o6nB 2ccmA 2re1A 3s4fA 1g8kG 1g20B 3salB 2h2uB 3lmwA 3kkfA 3kzpB 1l6rB 2i4cA 3q6dA 2qpuA 1yleA 1e54A 4bcuA 4b0pA 3hz2A 3kqaD 1uf3G 2vvfC 3v7zA 2fi4I 3m83B 2q69A 3oorA 3oheB 3edkB 3ab9A 3q09B 2rhpA 4f5yB 2ot4A 2e6uX 1o9iA 3w9tF 3q13A 4jdzB 3sjsA 1dx5K 3m5qA 1j0nA 3avwA 4ngnA 3ip7A 4jucB 1imeA 1qi5A 1i22D 3u48B 3fkhC 2voxA 4ovdA 1j9kB 2yb7B 3u4jA 4fyag 4k1cA 3d4gG 3gdcA 3cqoC 1cp9A 3nvnB 4pqiA 1ht9A 1j12D 4lmfD 4h15B 4n3oB 1k1xB 3iqeD 4k90A 2vmeD 3ossD 2oaaB 2whkA 4kppA 3ehjB 1xcbA 4jrfA 1mr8B 2b30B 4nsdA 2c9mA 1xmfC 1kapP 1qdbA 1m1jB 3gwzD 2q17E 2wqdA 3ubhA 1a25A 3njvA 3akbA 1n7uA 4bq4A 2gp7A 3pvoD 4n6fB 3f1vB 3bjeA 3fawA 1hkbB 4awnA 4nczC 2ykkA 3ldyA 2aerL 2c26A 4nuzA 3fzxA 2yfsA 4h0nC 4oy7G 3lagA 4celA 3co6C 2iwvC 3psrA 4bz4B 2iwaA 2pltA 1kuhA 2g42B 1uppE 1z6oC 2iicB 1u2vA 1sl6B 4hwvA 1ka1A 3tquB 2vnvE 1a0sR 1suiC 2zyhB 4ak7A 4ko1M 3wctB 1kdmA 2bamA 2iewA 4a15A 1yi7D 4lxoB 4fgcB 1g0hB 3gqfB 1c9uB 3f6yA 1jb0L 2y6jA 4afxA 3dbkA 2w66B 1blxA 3bwxA 1t1eA 2d00C 1gttD 4h7jA 3tkkC 2gsyG 3rhtD 2wp4B 3q9mC 1u5qA 1yaxA 3fd3A 3l1vA 4g1eB 3b7yA 2i8tB 1tkcA 2w87A 1m57G 1vldX 3a6zC 4kn9T 4sgbE 1tlqA 3ef2D 2w22A 1lw5D 3mcyC 2vn7A 3wu2c 4adgC 3qfrA 4ar9B 3cniA 1t6cA 3ur3C 1gxrA 3lzkC 5stdA 2wysB 3voaA 2e7fA 1kwhA 3vl7A 1hm9A 2hesX 4asmB 3qypB 1qlbD 2vqrA 1lomA 3zq4A 1uv4A 1qq9A 3q4gA 4j7mA 2porA 2y5pA 3rmkA 2je9D 2o6hA 4pibB 2il1A 2pooA 4i72B 2bibA 4b56A 4a9xA 3qhqA 1r64A 3c63D 3noiB 3e4qA 2ev5A 1d0lA 4kwuA 1exzA 4jbeB 2pmyA 3i57B 4eqbA 3mliD 1dykA 1e7dB 2zktB 3mk1A 2b9lA 1uy4A 3n0aA 3fozB 1r6vA 4b7bA 2e1pA 3bs6A 1hdhA 3lp9D 3er9B 1hqdA 3tb3B 2yn3C 2qt6A 4gncA 4dzrA 3powA 2b33B 2okxB 2pnyA 3kmnB 4i9xD 4hteA 4nt0B 3mhgA 2yhgA 1h0hK 2frhA 4hizA 4kc7B 3slpB 3s35X 3aluC 4gejI 2e26A 2xmoA 2ozbA 3eifA 3fz5C 1lpsA 2j5wA 2xc2A 2xxlB 4bloL 3g5cA 3s4yA 2ji3A 1b2lA 5enlA 4jx1A 4llsA 3o4yA 4n1iA 1ad5B 3in9A 4p0dA 1q39A 1uzjC 4bweB 3zdcA 4q0zA 3griB 1i9bD 3bfmA 3kh1A 3cv1A 2zl5A 1wc0A 2yhwA 4a4aA 3df6C 1ou9A 1iodA 3cneD 4ecwA 3pgbA 1t6mB 4apmA 2pvzA 4m5iA 3fgeA 4jk4A 3qe5B 1uisA 4lw9D 1jmjA 3k8kA 3dzmA 2c1vB 1lwjB 1bjqB 1i8aA 2z0jC 2xjpA 3wnkA 4i5nE 3or3B 1qmdA 3g27A 3zniA 4u65E 2scpB 3ptyA 4ce5B 2ix0A 4hhrA 1nzyA 3ttqA 2p6tG 2h1iB 3b55A 4a0pA 1brwA 1ekxC 3fz0B 4b4fA 1b4nD 4du6C 4awyB 3d8pA 2yeqB 2oznB 2wvzB 4nvrA 2id3A 2og9A 4ktrD 1c8gA 4afkA 3iucC 1v73A 3ouuA 4douA 3c7gA 1jn9A 1byfB 2ivzA 3chjA 1qi2A 1pg6A 1mveA 4kakA 3gukA 4f53B 1xhbA 3lorB 1nqdB 4bhvD 3ut0A 4by6A 4mgqA 3a68C 4j6rG 3sm3A 1jtdB 2o39C 2ddrD 1uvnC 2jdaB 4c16A 1o8gA 2vs7D 3c16A 2eroA 3pf3A 2xgpB 2taaC 4kxdA 3fvqB 2p5rA 1tjxA 4av3B 4k4iA 1kfqB 1smvC 3q7hG 3b4nA 3edyA 2cdpA 3na6A 4bb9A 4evhA 2xt6A 1y1aB 2nxqA 4l9oA 3dpgB 2xhjA 4ihbE 2c8sA 3fd2A 4ej7A 2essA 2dcjA 4ln1B 3t1gA 3hzbD 1x8kA 3attA 4olkB 4lqrA 2w4yC 2pplA 3k9jA 4jqpA 3ufkA 1cjyA 1gxoA 4m0kC 2zexA 3axhA 1uhaA 2rauA 3v5uA 4n2pA 1guiA 1de4I 2qp2A 3dt5A 2qtwB 1ga2B 1v7wA 3rqiA 4infA 4f9jA 3pp5A 4cc1A 2y3cA 2yv9A 3vj9A 3irhC 2vveA 3hjbB 1yvuA 4b5wA 3en8A 3goeA 1axkB 4hsqA 1x7iB 1up8C 3uowA 4mivA 4ii3A 4hpnA 4g62B 3pvhA 1wvmB 3gazA 2ea7A 4p99C 4kxvA 2plyA 3pe0A 3w7aC 2q1fA 3c5iD 1ji1A 3dssB 3aujA 2p1rA 3uvfA 1espA 6dfrA 3m0jA 2pjpA 4n1gA 3uf5B 3kstB 4lxzC 2rjiA 2es2A 1pk8A 4oyxA 1wwsC 4fl4I 3fybA 1p3hB 2fycB 3hm2G 3zr5A 3fvzA 1s99A 3toyC 1y6oB 1ee6A 3pddA 4cfrB 2i52F 4ibrA 1v3wA 4aylA 3bbyA 4axnB 4dktA 1m9iA 3rmkE 4l0pB 3lumA 3r4iA 2odiB 3fz3F 1o7lD 1j5uA 2e9bB 4e5uB 2wz8A 3wu2O 1b94A 2pc6C 3o4pA 4jzbB 4mrcA 4l75A 1h6gA 3b0xA 1mj2B 2fxfA 4ci7A 1bgpA 4lvnA 3r6qH 4lq0A 3wq7A 4k70A 3ipkA 4qu6A 3mkzA 4i2nD 2wm4A 2hcnA 2wgqB 1kitA 4n0oC 2xfvA 3t41A 2cmzC 2np0A 1qh4D 4jf7A 4ggqA 3r3tA 4m8dB 3sucA 3ck9B 4km6A 2ex5B 1hmwA 1c8nC 1nbwA 3bjuA 1nubA 3mq7E 4p57B 3etoB 1t5dX 4ghcB 4dnnB 3wfbC 3aqjA 3vtoB 2o4vB 2bh1A 4plrB 3u39C 3gflA 3uxfA 2i6hB 4iyqA 3jq1B 3nqnA 4m7kH 2gpjA 2owlA 1t9hA 4l03C 2wjsA 3auaB 1snnA 1rc8A 3v6iA 3dr2A 4nasB 4i9fB 4ag4A 2pyhB 3d7kA 4dk7A 4fa4B 2vmaA 3w5mA 3hiiA 1xzoA 3zo9B 3tb4A 2yknA 3fg3A 4c07A 2qm3A 2io4B 2btwA 2d73A 2oa8B 4lmhD 4hjfA 3ci0K 2jfpB 1qcnB 1ovaA 4mbzA 3gv5B 2zbaA 1w2mF 4immA 1szoJ 4dh2B 3u7zA 4ex8A 3ojcA 3r8yD 4ggfL 4aq0A 3vu1B 4aslA 4f3rB 3a8rB 4bxoB 2zzjA 4khoA 4it5D 4a42A 4fouA 2afbA 2r01A 4a6oA 4ic5B 4lfyB 4gx0B 3by6C 2c60A 3mzoB 4jsoA 3qwuA 3fcxB 1te2B 2wl3A 3jqxC 4bxoA 1q23A 3l2nA 2bueA 4mspA 4oa3A 1ff5B 1ck7A 4dlkA 2vl8A 1i9zA 4ac8A 1nnlB 2x49A 2oblA 2ygmB 1os1A 1t61C 1lkvX 3hbzA 1ug9A 1fyzA 4mkmA 2wg7A 3hlyB 1bagA 4lplA 2v9mA 3odtB 4p5fA 3wl4A 3rrwA 2xzvA 3rvvA 2e1qD 2iufE 2wkpA 4jo5A 4gkbC 2r16A 1k3iA 2wimA 4jclA 2durA 4mhvA 3tr5A 1nolA 4aw7A 4cidA 3qleA 2r2iA 3rq0A 3n9sA 3o3gA 3hl0A 1w5dA 1z29A 1bobA 3pz0B 3agnA 3smnA 1j83B 3hrdE 2vr0B 3iayA 4b0aA 3ia7A 1yfqA 1oupA 3c25A 3qfeA 3cvgB 1hdfA 3sqgA 3zypA 2zzvA 2cyyA 3ecqB 3bdvA 4jduA 4mboA 2i44A 3n91A 3qr0A 1fw2A 4g2sB 2vy0A 3rl5A 1nkqA 3exmA 3oqqA 3nurA 4efzA 3dedC 4luqB 4ecgA 4kh9A 1rk8A 2d4cA 3qc6X 2xhiA 1fxwA 2vn6B 3h4sE 2yocB 2wbfX 1yiqA 4k25A 4m7nA 3hfjB 1lvuD 3s6jE 2jalB 2zuyA 4ki8F 2qt7B 4nehA 1ve5A 3nngA 3ktbD 4g01A 2g0yA 1t0iA 1w07A 2cn3B 2xvtC 2bq4B 4mr0A 3dxpA 2qimA 3e79A 2xqoA 1wmeA 3b2zF 1eggB 4nhdD 2w3oA 4mz0A 4petA 3jtxB 1ohzB 1uhnA 3ak5B 2xqxA 1zud3 3s4lA 3lybB 2eufA 4bgbA 2xbfA 2q1cX 2pr7B 2w1uB 1ru4A 3dasA 1dmuA 2az1D 1qlsA 2gf4A 3g0kA 3fhaA 2aprA 3qmnE 2wynA 2bo2A 3km5A 1qhdA 1tw8A 2qeaA 3dd4A 4eoyA 2vyoA 2aorB 3bvcA 4plmA 2fi1A 3h36A 1v0aA 3gg1B 2ricC 3sonA 2gseA 1wy9A 1pocA 1glgA 4ozgC 1y10C 4npkA 2v73A 3pk0C 3trpA 3pf2A 2vptA 1dfmA 1nw1A 1ag9B 4gsqA 1rdrA 4kkiA 4o5mA 2z6iB 3ectA 4aq1A 2d3dA 4mlzB 3bx1C 3p6bB 3v64A 3haiA 4g26A 2o18C 3achA 4cu9B 2qngA 2zahC 3m1rD 3p2yA 1ydyB 4mmhA 4l2eA 2nvoA 1wadA 3lvtA 3aagA 2qltA 4apxB 3mt5A 1cvrA 2jcgA 3sh5A 3u43B 3kg6A 4mlcA 1nrwA 3db7A 4jglA 3ju7B 4mzaB 1eptB 3qqzA 2gxsB 4mixB 4ku9A 1z0bA 3kqgA 3e3rA 4jwpA 3cjyA 2vmhA 1srrC 1gn1F 1wkyA 3bowA 3q3qA 2yayA 2rldC 2i1qA 4iu2B 2nxpA 4mkfB 1dl2A 1mnzA 2wfkA 3qtaA 3asiA 2xwgB 2hnfA 5chyA 2dsoC 2hq8B 3zukB 3ukgA 1f4nA 2e85B 2bngB 3hjrA 3lcpD 4b7uA 4ptfA 1o5kA 4d90A 3wmwB 3kptB 3lnpA 3rzaA 3iprF 1r17A 3s55D 2qv6A 1yu0A 1y1xA 3hx6A 4iu2A 3zm8A 4ht4A 3votA 3b40A 1f0oA 2osdA 2jbhB 1of3A 2eabA 4i9wA 1lqvB 2snsA 3hciB 3vm5A 3pgsA 3b3dA 1p49A 1c1yB 4eg9A 3f8hA 2fpwB 3djlA 4hvkA 2ddfA 2arhA 3kmvE 3wmtA 4p5eB 3ltlA 4mn0A 2x7qA 3ojyB 1ng0C 1nenB 2xmrA 1zmrA 3njhD 2eslF 3ffkA 2wfrA 1fwxA 4gz9A 1k6sB 1g5cA 3godA 1sumB 3v20A 3lwaA 3u16B 1fsuA 1ej8A 4dokA 3k28A 1h30A 3fspA 2iunB 1e7mA 3whnB 1kp4A 4il1A 3by9A 1h6xA 3k5mA 2iiiA 1w3bA 3im9A 4hojA 3oyrB 1mu5A 1tn3A 2y8kA 1j1tA 3mseB 2g8sA 1bf2A 2x8hA 3dtnA 1oflA 1m5oF 2tbvC 2pagA 3dzcA 2i7aA 3s6pC 3eqiA 4uojB 3pe7A 1nl1A 1tvgA 1n7sB 4ll5A 4lp7D 1j24A 4aejB 3zzrA 4ollA 1zdeA 4dsiA 1f8vA 4m5vA 3mdoA 4be6B 3ntlB 1zobA 3qdnA 2b50B 3svlB 2wiiA 3p4gB 3srgA 4iaiA 3us9A 4nwoA 2v3tB 3ibzA 3r5xD 3kezB 4n5xA 1u94A 3e03A 3n1uA 2w27A 2ydxA 2xecB 4iauA 1y7uA 4fzmA 3pgvA 2yf1A 1vsiA 4c0kA 3n9kA 4b60B 4kpoA 1tadC 1rq5A 3gw6F 1je5B 1heiA 2f1wA 3kaaB 3tbdA 2gskA 4awdA 2q04A 2uvpC 4lt6A 2y3nB 1uocA 2h9dD 2cftA 2h0bC 1su3B 3rrvB 2omxA 2g8kA 4ovyA 2ydpA 3ppvA 2xonL 4h2aA 2ww8A 3eu3A 3s82B 1hcuB 2xrcB 4g97A 2a11A 4fhpA 3qsjA 1xkdA 1e3aB 4nhfF 3vppA 3gbhB 3riwB 2cy5A 1pmjX 2ezvB 3mvsA 2bf0X 2bkoA 4i9xB 2ygpA 3nsjA 1jhnA 2imwP 4i4tA 1ex0B 4iejA 4m00A 2bw1K 2nq6A 3o78A 4kywA 3afgB 3d1rA 2g18D

Independent evaluation set

1g9uA 1ayoB 1dctA 2c4dA 1mpxC 2wyfD 4hroB 1upsA 2c5uB 3l8yA 4q51A 3h6gB 2c3oA 3p85A 4fsoA 2pqxA 4hxbL 2clpC 3flrA 4m6xA 2jg9D 3qm2B 3htlX 3cu2B 4jjjA 1ul3B 3ge4I 3bcfA 1kkmB 1rtp2 1ys6B 1ydnC 3wd9B 4h2iA 2fkcA 3w7uA 2dbxB 1g8hB 2ejnA 4abtB 4g1eA 2ozbE 3ovsA 2bs9E 1ti2A 4fjiA 3ai7G 2gftB 1xxmD 3ayfA 4ln6G 4linF 4o6nB 2ccmA 2re1A 3s4fA 1g8kG 1g20B 3salB 2h2uB 3lmwA 3kkfA 3kzpB 1l6rB 2i4cA 3q6dA 2qpuA 1yleA 1e54A 4bcuA 4b0pA 3hz2A 3kqaD 1uf3G 2vvfC 3v7zA 2fi4I 3m83B 2q69A 3oorA 3oheB 3edkB 3ab9A 3q09B 2rhpA 4f5yB 2ot4A 2e6uX 1o9iA 3w9tF 3q13A 4jdzB 3sjsA 1dx5K 3m5qA 1j0nA 3avwA 4ngnA 3ip7A 4jucB 1imeA 1qi5A 1i22D 3u48B 3fkhC 2voxA 4ovdA 1j9kB 2yb7B 3u4jA 4fyag 4k1cA 3d4gG 3gdcA 3cqoC 1cp9A 3nvnB 4pqiA 1ht9A 1j12D 4lmfD 4h15B 4n3oB 1k1xB 3iqeD 4k90A 2vmeD 3ossD 2oaaB 2whkA 4kppA 3ehjB 1xcbA 4jrfA 1mr8B 2b30B 4nsdA 2c9mA 1xmfC 1kapP 1qdbA 1m1jB 3gwzD 2q17E 2wqdA 3ubhA 1a25A 3njvA 3akbA 1n7uA 4bq4A 2gp7A 3pvoD 4n6fB 3f1vB 3bjeA 3fawA 1hkbB 4awnA 4nczC 2ykkA 3ldyA 2aerL 2c26A 4nuzA 3fzxA 2yfsA 4h0nC 4oy7G 3lagA 4celA 3co6C 2iwvC 3psrA 4bz4B 2iwaA 2pltA 1kuhA 2g42B 1uppE 1z6oC 2iicB 1u2vA 1sl6B 4hwvA 1ka1A 3tquB 2vnvE 1a0sR 1suiC 2zyhB 4ak7A 4ko1M 3wctB 1kdmA 2bamA 2iewA 4a15A 1yi7D 4lxoB 4fgcB 1g0hB 3gqfB 1c9uB 3f6yA 1jb0L 2y6jA 4afxA 3dbkA 2w66B 1blxA 3bwxA 1t1eA 2d00C 1gttD 4h7jA 3tkkC 2gsyG 3rhtD 2wp4B 3q9mC 1u5qA 1yaxA 3fd3A 3l1vA 4g1eB 3b7yA 2i8tB 1tkcA 2w87A 1m57G 1vldX 3a6zC 4kn9T 4sgbE 1tlqA 3ef2D 2w22A 1lw5D 3mcyC 2vn7A 3wu2c 4adgC 3qfrA 4ar9B 3cniA 1t6cA 3ur3C

1. Co^2+^

5-fold cross validation set

3i11A 2vc7B 3thhA 1t31A 4n7qA 3iuhC 1v29A 2xtsC 2xinB 2xjmD 2b3kA 3schA 2hn1A 2ps9B 1l8xB 4gjyA 3rmuD 2fuaA 3n5fA 1tu4C 1wmpB 1rxtC 1b59A 1stgA 1cobB 4g3qA 3tk4A 1rrkA 3e3hA 4cv7A 1t5kA 1fsqB 2claA 4q0uA 3v9uD 2icvA 2zu9A 3mf3B 1vlxD 2uzpC 6celA 1uv5A 3g5pA 259lA 1c4gA 4aq4A 2fqoA 2amxA 2zc1A 2bo7J 1jn1A 1r0hA 1k1eE 2bdiI 1of8B 2ew5A 3ut1A 3nzhH 3ojkD 1rv8D 1gwmA 2os3A 1vz0D 2z06D 3l0sA 4jh5A 3zr9A 4askA 1rmqA 3r4qA 3m4zA 1fofA 3gobC 2xdmA 4m5cA 1rqeA 4jbhA 4ft8A 3dwcB 1r8kA 3d03F 4gprA 2j3zB 2xvzA 1hgwA 3i8vA 2parB 2xphB 1m38B 4jz2A 4jkaB 2c79A 4eraA 1bk5B 3alqS 4i2gA 2yyeB 3bd5B 3ttbA 3r61A 2wyrH 3h9aB 3kolA 4litA 1lnaE 1qxyA 4metA 4jkyB 3w45B 2gruA 1xmhB 4nncA 3pniB 2y8uB 3vxmE 3lmmD 1y6vA 1a0eD 3mgpD 3pjlA 3oceA 2ddsA 3m5aA 3sxxA 2ehdB 4afoB 3hdiA 3s5hA 2f7nA 3nvlB 3ggdA 2f0aB 1wn1B 1gqjA 1jymA 2dfiA 4ekdA 3gqaA 2effA 2y3bA 4iwwB 1fa6B 3p0fA 3tghA 1h0nA 4eroA 3nykA 2gliA 3mcrA 1iabA 1ukwB 1qreA 1scsA 3ka9A 2ywrA 3tw3A 3tr3B 2vosA 2xdbA 2j4jF 3khcB 4magA 4b5wA 3ta5A 2qb7A 2prqA 4nuiA 4cv4A 1r6xA 2oi6B 4iynB 4n8mA 3rgtB 2f7vA 2q6qB 3ivuB 3h2wA 2opcA 3uc3A 3si8A 2jf5B 3krgA 4njqB 1xrcA 1nr5B 1zjcA 3cunB 3mz7A 3qq7A 3s8kA 4as7A 1fx7A 3isqA 2r5vA

Independent evaluation set

3i11A 2vc7B 3thhA 1t31A 4n7qA 3iuhC 1v29A 2xtsC 2xinB 2xjmD 2b3kA 3schA 2hn1A 2ps9B 1l8xB 4gjyA 3rmuD 2fuaA 3n5fA 1tu4C 1wmpB 1rxtC 1b59A 1stgA 1cobB 4g3qA 3tk4A 1rrkA 3e3hA 4cv7A 1t5kA 1fsqB 2claA 4q0uA 3v9uD 2icvA 2zu9A 3mf3B 1vlxD

1. Cu^2+^

5-fold cross validation set

4dyzA 1id2A 3fboA 3bktA 3ppsB 3epvB 4alrA 3gyrE 1rk5A 1ibyC 1pzsA 3n7eA 1m57B 3tyhG 4oy7A 3g5wB 1jerA 3tasB 3nbbB 3aasA 1ksiB 2y9xB 3k7rE 3de8B 3e6zX 1odbE 3mztB 2wq8A 1asqB 3mn0A 4hd6A 4hcfB 3t6qA 4lsyB 2fouA 3zjaA 2ztkA 4e4zA 1cc3B 2xmkB 1h1iB 1s4cB 3awuB 4oakA 2b7jC 2fk1A 4mntA 3higA 2uwfA 2yc3A 2xz4A 2c9qA 3od3A 4h7lB 4eirB 2xjnK 3iudC 1t16B 2j5wA 3wa2X 2xlaC 2voxA 2zcvA 4flmB 3gdcA 2iwkB 2e46A 4o65A 3b1jA 1thoA 1r5aA 1bugA 2oxiA 4phzI 2fu7B 1ov8B 3umkA 4hvoA 1gy2A 2jlpD 1a8vB 1aqpA 4j3rA 1lcfA 1gmwA 1v6pB 2gimC 1zm5A 4lejA 4n3uA 4phzC 3zx1A 3pgbA 1s1qA 1xmeA 3sb8A 1zxiB 2yevD 1sddB 1ukuA 4jfnA 3ziyA 3u52A 3sesA 1iaaA 1oacB 3zudA 4kavA 3e0iA 4nunA 3lzqB 3w6wA 2aqsA 4b61B 3tu6A 3hhsA 1ekjG

Independent evaluation set

4dyzA 1id2A 3fboA 3bktA 3ppsB 3epvB 4alrA 3gyrE 1rk5A 1ibyC 1pzsA 3n7eA 1m57B 3tyhG 4oy7A 3g5wB 1jerA 3tasB 3nbbB 3aasA 1ksiB 2y9xB 3k7rE 3de8B

1. Fe^2+^

5-fold cross validation set

1cjxA 1dv6L 1e5sA 1ey2A 1fz0A 1gp6A 1h5zA 1h7aA 1jr7A 1mmkA 1mpyA 1nf4I 1oaoB 1oqbF 1os7A 1rrlB 1rrmA 1rsrB 1smwA 1sp8D 1unbA 1w06A 1w69A 1wa6X 1y0zB 1yuzB 1zt5A 2a1xA 2bq8X 2ehzA 2fctA 2fdjA 2gcuC 2ivpA 2ji2A 2pt2A 2pxjA 2qh1B 2r1lA 2vw8A 2wboA 2xgfC 2yc0A 2yu2A 2z90A 2zi8A 2zo9B 3ak9A 3aqjA 3ayzA 3bz2A 3e1qI 3fg3A 3gzyA 3jskN 3k9zA 3lfmA 3mguA 3mz6A 3n9mA 3nl1A 3obzA 3ovpB 3pl1A 3r60A 3ussB 3veoA 3vmhC 3vshD 3wr9B 3wsiA 4b2oA 4bmqA 4f2zA 4g33A 4ghcC 4gulA 4h3eA 4hr4A 4hz1B 4il6D 4j1xC 4kbzA 4kwjA 4m51A 4naoA 4nm6A 4o6jA 4oj8C 4ou9A 4qddA 6prcM

Independent evaluation set

1cjxA 1dv6L 1e5sA 1ey2A 1fz0A 1gp6A 1h5zA 1h7aA 1jr7A 1mmkA 1mpyA 1nf4I 1oaoB 1oqbF 1os7A 1rrlB 1rrmA 1rsrB 1smwA

1. Fe^3+^

5-fold cross validation set

1gvgA 2rg4B 2ohiG 2w8sC 4aq6D 4hbhL 2csgA 1ykpH 1k70A 4qqwA 3ldbA 4fm4C 2gbwE 2iuwA 4k19C 2jblM 3l0pA 4m6xA 3dhiA 1nmoF 3bvlF 3hh8A 3d19A 3eqeA 4kkzD 1w2aX 1z02A 3nnfA 2gm6A 2jb4A 3fpvD 1j3qA 4rxnA 3c25A 3qgkI 1dmhA 1mtyE 3qhbB 3fvbA 2vunD 2q4aB 3wfdB 2fjcB 4d8gB 4ergA 3fm6A 3o1vA 1xm8A 3ivdA 2uyuA 2ydeA 4mcwB 1rsvB 3aalA 3ufkA 4iwjH 1o2dB 2vzbA 3t81A 2biwB 1auiA 1aorB 3bv6E 1tjoA 4jo0A 4f9jA 2pybA 2gp3A 1z6oW 3gteA 4ewaA 2yxoB 2ji3C 2xv1A 4ha0B 3fmrA 1kmpA 3lmcA 1nx8B 1shrB 2z36B 3zpoA 4qlwD 1xvxA 1ltvA 4kvqA 2puzA 4mlmA 2c6rA 4b8yA 4olsD 1lnbE 3maoA 1sumB 1y4tA 4mhuA 3qc3A 3ks5B 4ku0D 3cpxB 3fvzA 4nd8D 4nubA 2amuA 4f0lB 3u9mE 3li2B 3fe5A 3r2uA 2bi4B 1oquC 4e4hB 3zk4B 3bxvA 2yeqA 3cf4A 4huzA 3l87A 3s6bA 3tlkA 2clbC 2itbA 4b28A 1warA 2yjkH 2ogiB 4qdcA 1nf6F 3bxdA 3wsfA 2w16B 2phmA 1y07C 1veiA 2b5hA 3h9aA 1y56A 1yuzB 1y67D 3dbyT 3oghB 2hk6A 3vsiB 3qy8A 2j2fA 3vcpA 3o0fA 3vtoB 2q0jA 2innA 3qy7A 1no3A 2zziB 2gyqA 1rcwB 3qxbA 1bszA 2h0vA 2vhlB 3vthA 2xmoA 1zgnB 1t71A 1cojA 1vljB 4aiqA 2p4zB 2i9uA 3e74B 1tmxA 3pu8B 3d3lA 4ne0A 3kt4A 1frfL 2wl9A 1q0oB 3qr7B 4jydA 2c2fA 3wpmA 2fiyA 3qfmA 3bwwA 1xsmA 3htnB 3lm4D 3v7pA 2yivX 3ktcB 2iw4B 1wraB 4j5iB 2rdnA 3perA 3saoB 3ib7A 4ac8A 3zk3A 3hc1A 3hq0A 4fagA 2bjjX 2pq7A 2wluA 4cybJ 3tmbB 3rf7A 1sqdA 1gp5A 1gupA 3pqiA 3vv9B 3nteA 3omiC 3dcpA 1vrbD

Independent evaluation set

1gvgA 2rg4B 2ohiG 2w8sC 4aq6D 4hbhL 2csgA 1ykpH 1k70A 4qqwA 3ldbA 4fm4C 2gbwE 2iuwA 4k19C 2jblM 3l0pA 4m6xA 3dhiA 1nmoF 3bvlF 3hh8A 3d19A 3eqeA 4kkzD 1w2aX 1z02A 3nnfA 2gm6A 2jb4A 3fpvD 1j3qA 4rxnA 3c25A 3qgkI 1dmhA 1mtyE 3qhbB 3fvbA 2vunD 2q4aB 3wfdB 2fjcB 4d8gB

1. K^+^

5-fold cross validation set

1a49H 3c33B 1hpmA 1d7uA 1m5hE 2wmeC 2fcaA 4h5jB 1jjwC 2qv6A 1r64A 2bkpA 4jayC 2oecA 1gkzA 3hw9B 3fd5B 2fxiA 1jdbF 1krjA 1ax4D 2ibwC 2j41D 1ud6A 1jdbK 2whvA 2pa2A 3zd9A 1fpiA 3fgoB 4it1C 1dtwB 2a0qD 3ss8B 1wcfA 1dioA 1dtwA 1ttqB 3crlB 1gzgB 4a0mA 1nhiA 3umoB 2xptA 4twkA 1w22A 2a6vA 1gupA 2b5wA 3s3xA 1me7A 2zxeA 4m3pB

Independent evaluation set

1a49H 3c33B 1hpmA 1d7uA 1m5hE 2wmeC 2fcaA 4h5jB 1jjwC 2qv6A 1r64A 2bkpA

1. Mg^2+^

5-fold cross validation set

3cmeT 3zvlA 2hx1C 2hv6A 1x8bA 3tiqB 3doeA 2y87A 2c5uB 2xj9A 1rlmD 3qxcA 3mtcA 2c3oA 1ys7B 4g61A 2dgnA 4hubL 1we3A 3tavB 1tt4A 4okzD 2fkzD 3fqxA 3dkcA 4ay7B 3i0oA 4ifzA 2yniA 3lopA 1bwvC 1h74B 4dfdA 3nemB 2hkjA 3f79E 1w78A 2q0yA 3mg8L 4ffoA 4qjlA 4fwiB 2gjkA 1g8hB 3s89C 1vx9P 3v23Q 1qu2A 3cmwA 1z72B 1wpvB 4d09B 1ig5A 4kxwA 1aqfH 2fcoA 4qcr2 3fkqA 3ct7E 4b3tN 3tw6D 3cq3D 3q45A 4hnzJ 3ahcA 4kj53 1m3uI 3qfvB 1m1bA 3oevK 2q5zB 4klnA 1amuA 3jz0B 1svwA 4c7yA 2pk0A 1jd2G 1ovdA 3ey9B 1uutB 2whiE 2i5rB 3h87A 2r8eH 3ejaA 2vzbA 3vnpB 4qcpD 2ihvD 3pdeD 2rcyA 4c0lA 2vbvB 1ne9A 4obbA 3w9tF 3vatA 2hw5D 4as5B 2zrzA 1kd14 1t5jA 1gljY 1cmcA 3ig5A 1urbA 2p06B 4iokB 4f6tB 1q19D 3f1eE 3a7rA 4qcrW 1ryaB 3u4mA 1n0hA 2gx2I 1v14A 3es7B 3fefC 1wpgA 1efkC 4bvrB 1iw7O 3ld0U 1fiuC 4hb4A 3kwsB 4nx8A 3knhO 3eywA 3zvhB 3a4wB 4mzuB 2a69C 3bhtB 3cbtA 4q04A 3h7vA 3nm7A 3hdgA 3ohzF 4nkbA 2vn2A 1dakA 3knhF 1nj1A 4hxgF 2y4mB 4dtfA 4bpwA 4qcz3 2quiA 4qcnH 1q6oB 3c4qB 3lasB 3o5tA 1qhyA 4eo7A 3vayA 1v5fA 2qojZ 3na8C 4qcrN 2yfnA 4qbyI 2g8hA 3ed6A 1cw0A 3c22D 2a9fA 2e67B 2x3jA 1xmqJ 3bwlB 1p5zB 2oiwC 4ltzA 4qcr8 2aerL 4tq4D 1nlqE 2bhdA 4fvuA 1znoB 2pmqB 4dpvZ 3n2nD 4ev0A 3ocvA 4ariA 3k9fC 1h56A 2yevD 2yvmA 4dkwB 2a87A 1sojD 3uqeA 3pdtA 1iruU 1q9sA 5xiaA 1ka1A 1fjgL 1it8A 3d6wA 4ip2C 3koaA 1mowA 4gp7A 3l23A 4cglA 4as2B 3akmD 4pyoB 3au9A 2npiB 2pgoB 3etjB 2o1oA 2icpA 2yi9C 1d2iA 4mpoB 3cmeB 1jylC 4qcr1 1svmC 4nb4F 2px0H 3mf4B 4nvvR 1n32D 2uu7I 2vjlA 2zxcA 4if4D 1b8cA 1gtvA 2zanA 2xhvB 3t5pL 3wgvA 2y4aD 1d9dA 4h0iA 2fbxA 2as8B 2bphB 4nvvG 4gzkA 1ow2B 2b1qA 1miwB 2ad5B 1e1zP 3nvsA 2yxhB 3e48A 3rimC 3v2uD 2hwgA 4bkmA 1fx4A 2ji6B 2g0wB 3fvyA 2j1nB 3mesB 4e6mA 3f1fS 2d2fA 3f61A 3deqD 3r76B 2x7aI 3sduA 2f9rC 2q40A 2amcB 3f85A 2qvrA 2ejwA 1ec9D 4a48B 3v5wA 3obkH 1pg4B 4qczX 2zyrA 2o4gA 2ag0A 1zotA 3v8eF 3s1aA 2dcnB 4q3bD 3qlzB 4cogC 2r42A 3qe6B 3mcoA 1y9aC 4hzdA 2xsqA 4qctO 3fd5B 4nv0B 3o2eA 4kwuA 1rkvA 4hqoA 4amsA 4qczU 3dgtA 3fwsB 3hiyA 2h1cA 2f43B 2dkgA 4kgmA 2yp1C 3sz4A 3en9A 1t3nB 4b3tG 3f1eX 3t5tB 4dzhA 2g25A 3cinA 3h7cX 4miwA 1ytuB 4c3iB 4knhB 2v54B 4be1A 3s9fA 3gnzP 2hvqA 3qy7A 4a8mQ 4g6hA 3dxjD 1q3uA 4f8eB 1r2rD 1sxjD 3k57A 4njiA 1nbmC 3r9xA 1xxxC 2oekA 2fp0A 3wqmA 2aqxA 4q1vA 3g1bB 3kni0 1kxgA 4flxA 2vf7C 4a01B 2yhgA 4lrzD 4dn1A 1musA 2hsjD 2p3nC 3r4cA 2bz0A 1rfqB 3c8vC 3s7zB 4dmzB 3f1eT 4kemB 3i6tB 2jd4B 3cifB 2feaA 2ggeD 2d5fB 1eyzA 3gieB 2aekB 4m83B 3mp3C 3c5pD 2j3qA 1l0oB 4ncaA 3u4fB 2iutB 3bwvB 1xdpB 3ilfA 4b61A 4qcn4 1g8xA 3fj4A 2oi2A 1z2oX 1golA 2ix1A 1zbuB 1su2B 1mc3B 3vpnA 1dekA 3wbzE 3ae0A 4qctV 3bl5E 2oa6D 1tq6A 1g9xB 4pl0B 2npnA 3e3zA 1q3bA 3eueA 3djbB 1y9iB 3hnmC 2qenA 3mmnA 2x98A 1sgjC 3zthA 3kr4C 4kj2N 1jgtB 3m01A 2zgzB 2zl5A 2pnqA 2bx2L 3e4dB 2ddtA 4bl0A 4bjrA 2pi4A 1o4zB 4dn5A 1rdfE 1ar1B 2z2pB 1dqnA 4qhtC 3gn6C 4db8B 3smlA 1ewkB 2x4dA 4ecwA 2wvlA 2y7fD 4j6wA 2p4pB 2wtzB 3mx3A 2x03B 1twyA 4i5qA 3k8kA 4hvjB 4la6A 4hchA 1zszC 3f1fI 2ofxB 1xqaA 3oi7D 1ta0A 4b3mI 4m30A 3kkoA 2vhxF 3wrxC 4iedC 3jvtB 3e2dA 4b3mB 3b8iF 1h7qA 3knkZ 2a5zC 4kj9D 2qm1B 1vtnC 8iciA 2qv7A 2x0qA 3ip4B 4m5nB 1u3cA 3rmwA 1u7pA 4nstD 4awyB 3s14A 3w40B 3tepA 2wvaX 4qdgB 3rfeA 3t1rC 2jkgA 4ggmX 4gu5B 2zscA 4kj6U 1k77A 1k68A 2isiA 4bbjA 4q86H 3u7qD 1nziB 4hubN 3ii9C 1wywB 3shxA 4kiyV 4gbfA 1u02A 1rkqA 3o98B 1bl3C 2d0bA 2j7nA 1iruY 4jejA 3u0oB 2i71B 3ozgA 1nmpE 3bznA 2p5eB 3ef1A 3ktnA 2ia5H 2d32A 1y37B 3r7wC 3na5A 2j5vA 3kd3B 3q6jA 1j7lA 1yunB 3mmhB 4aq4A 4en4A 1obgA 2br3F 2v4oD 4omfA 3k5hA 3ivsA 1s4eG 1nrjB 2a41B 4nmuD 2b8jB 2xkbI 2huhA 2pa4D 2dsyB 4cc7M 4fh5A 2xt6A 4jasA 1w23B 4idnB 3swiA 1g69B 2xspA 2f1fA 1gqcA 1wzcB 2xdgA 2o3bB 4oz6A 1w85C 4dscB 4l8nA 3attA 1v26B 4j03A 2q3wA 3kx2A 3ufkA 4e7pB 3wxmA 2gslF 3reuA 2qndA 3e4oB 3pmgB 3vytC 2jf5A 2r5wB 13pkA 1jlsB 1ga2B 4qjbA 1r0zC 2yzjC 1mumA 3hwx1 2alfA 1gkbB 3n84C 1w0nA 2o35B 3tvaA 4j13B 4hppA 2hawB 2pp3C 4m4qA 1htwA 3q7eA 3o3fA 4jn9A 3mwbB 1hbnF 2v9pA 1xd9A 4l80C 4k99A 4gedA 3dlzA 1xbtA 1qb7A 2od0A 4ii3A 2qlxB 1wvmB 2o8dB 3ozxB 1tqyC 3fp2A 3fe4B 2q0eA 1knyB 2plyA 1f5nA 4lsbB 3q46A 4nclA 3rv0B 1q9kB 2i4oA 2pjpA 4ajrA 2vrnA 4un4B 2cjeA 1pfkB 4e2pA 3fnbA 2hfzA 2uxqA 3pt1A 2pzaA 3ssoD 3l8fA 1vrpA 2ajrB 3r2uD 3eg7B 3hm2G 1yfrB 1wuuA 3klcB 4oeiA 4f5dB 3gcmB 1yw0D 2gmsA 4m33A 3gsiA 2awoA 3aq0C 2ppqA 1yvhA 1esnA 3uoaC 3kzqB 4azwA 3uzrA 1ze1B 2bmuB 2pywA 1q4rA 1dgmA 3n3tB 3ffuA 3wcwB 4mhxB 1qpbB 3vc2D 1v8vA 1i7qA 3aqbD 2gfqA 4fk1D 4bwrA 2qvhA 3onnA 3zkbD 3qr3B 1i3mA 1wbbA 4ijxA 3qf7A 1ouoA 3hfwA 2okvB 3v6jJ 4cvoA 4i27A 3bpdF 3gveA 4brsA 3hb0C 4fmaB 4i3mA 3ob8C 4hjhA 4ak6A 1lnzA 4g7hP 2ik0B 1y8aA 1rifB 3w2wA 3oghB 3hrdA 3tawA 1s5jA 3vkkA 3q1oA 1nr9D 3pegA 4l6aA 3r10A 3sucA 3gf0A 2dh4A 3zxsA 1t57B 4e8lC 1rvcB 3qy8A 3pu9A 3o8rA 3rplB 3uplA 1p18B 3vzxB 3knk5 1rzrA 2fglB 4eoyC 3c6aA 4lemC 1yxoB 3nzgD 1wvcA 3n4fA 1gqyB 4fppB 3rt0A 4dgtB 4hv6B 4c12A 3cawB 1nuiA 3ohaA 4fk9A 1xrjB 2go7C 1vx8H 2xtoB 3tp4B 1n67A 4e6eA 4i8eX 3vh7E 1h7uA 2izsA 3dd2H 1iruC 2qgyA 2x6vA 2iqqB 3zo9B 3n1gA 3zx4B 2fueA 3bqbX 3q15D 2xcwA 3f2bA 4qcvY 4l3aA 3bm4B 3auoA 2v5kA 3vczB 2fq1A 3jysA 4jbtA 4immA 1fhvA 1q3qC 2iyyA 3orqA 4ktzA 3r3sB 3njlA 1x07A 3slrA 3ppiB 3wewA 4j5iF 3c9uB 2gqsA 3i8bA 3gxzB 3ulkA 1yl5A 3ksqB 3ombA 4q4aA 3qvqA 2rioA 3t6rA 3dylB 4o2wA 3fdgB 4lpsA 3qqyA 1w49B 3bk5A 3aqlA 3ez6B 3exfC 3e5zA 4f0qB 4m69A 3da3A 1iwlA 2oy3A 3w6nB 1wnoB 1nulA 2flcA 4b3mQ 3ephA 2plsI 2aruA 3v94G 3uulA 4osqA 4hubZ 2x1aA 4ag5A 2om6B 3q22A 3m6zA 2p97A 3vpbB 4kegA 1w1wC 3kkiA 3gahA 1o6tA 3hs0F 2yk0A 2w8dB 1ohfC 2vu9A 3wryB 3b1vA 3fvdB 3gocA 1os1A 3iceC 4kavA 3ie7A 2r25B 3s5fB 3ic4A 3bs1A 2ewgB 3cvjA 2hpkA 4fvaC 4lplA 4jx0B 1zn2A 4k9qA 1yu4B 1a77A 4b3tC 2jihB 2z4tA 3vzdC 2hfkA 2prnA 1izcA 4izkA 4i45A 1oh9A 3b7wA 2gwcF 2wkpA 3rf6B 2yfdA 3rgnA 3viuA 3pffA 4pu5A 3mqyA 3i9gL 2reuA 4e89A 3gqcB 3e5hA 2eb6A 2iciA 2q66A 4cidA 2ejqB 4g75A 1xtjA 4kjgA 4cltA 4l0jA 4ml9A 3ucwD 2j4eD 3rcyF 3lgyA 4ja9A 4cyjB 3pnlA 2yqcA 3d19A 1fi1A 3shsA 2nogA 3rnlA 3cmeC 2r7dA 2b0tA 3fr8B 2e89C 4b3mM 2ibpB 4at8A 1k4iA 4ocpA 4ba9F 2q5lA 4lgyA 3btxA 3q4oA 3raeB 4lnjA 4kysA 3adcA 3vkgA 3ugvF 3nl6C 4knvA 4p02B 4a35A 4f8bB 2c71A 3fpaA 1fxxA 3vr6B 4mn3A 1xd3A 3t99A 4p32B 2c43A 4ogeA 4afyB 1qr0A 2wagA 1t0fA 3jvvC 2o1xB 3f1fK 4iefH 4anjA 1f6tC 4kh4B 4fi1A 4r02V 4nnaA 2v9jE 1ka2A 4nehA 3to3B 2fa9B 1do0B 4ix5B 2z51A 3l9bA 4olsD 3hwoA 2hpiA 4fm9A 1vc9A 4jrnA 3epsA 3chmA 1repC 4h9dB 4g7eB 2f7tA 3qqvA 3ddvB 1vr0A 3vb9B 3dtyE 4ep4B 2i6kA 3cisB 1t8qB 2xs4A 4kftA 1hbnA 4hvyA 3f1yA 1n52A 2jd5A 2c2jA 4jzvA 1iovA 1inpA 2hmaA 3hujH 2yl6A 3c8cB 2zpuA 4gi2A 3qurA 3hy6A 4py5A 1gs6X 4il8A 4kp1A 4ex4B 4orkA 2qdfA 4bjuA 2vosA 3feuA 1n2gB 3auyA 2ox6A 3dv9A 4oxdA 3cuzA 2bonA 2hmcA 2pyjB 3l12A 4jndA 1gxbD 4bebB 1v98A 3rvpB 3fxgE 1qhaA 3bc1A 2b06A 1m0wA 3t2dA 1waxA 3htwA 2xzwC 2ouxA 4odjA 4kvaB 2ou7A 4m0lC 3eafA 3chlA 4iynA 3tzfA 4kjcT 4gwiA 3sy8B 1u5rB 2uagA 4hizB 1m74A 1vbgA 4eenA 3kb4B 2is4A 4czkA 3ea0B 1gsaA 2wcjA 2x5fB 1wc1A 3rgwL 3qe5A 3shqA 1wywA 2ddxA 3k1uA 1yrbB 1rypH 3g3nA 4oojC 3fdoA 3tebA 1qlgA 2w5aA 3ir2A 4dbhA 2onsA 1nhiA 1yvpB 2a5yC 4twkA 3b9tC 3m7kA 3hs0I 3h70A 4f0yB 3mbiA 2wl9A 3opkC 4cejB 3vthB 2xd4A 3ovbA 4ku9A 4cnsD 4l39A 3c15A 1cx0A 2bgwA 1hk7A 3iilA 3fr6A 3akeA 3no1C 3p96A 2vd9A 4fmmB 1fvuD 2qs8A 3n6qD 1tw1A 4oauC 2fyrA 2a0iA 2eb1A 3st8A 4dmbB 2z4sA 2cjaB 3wcaB 1khzB 3b1rD 3wagA 2eh3A 2qt0A 2guhA 1y23B 2h28A 3iukA 4fypA 3umbA 3bp1D 3nx3A 3c9hB 3kd5E 1sh3B 4d26A 3d2fA 4n0nA 4n5vB 1zshA 4q7fA 3wkyB 4l87A 2xamB 3k4zA 4m9qB 4eruA 3kylA 3v9xB 3kb2B 3tdwA 4hubY 1id0A 3t1oA 2ddoA 4kg0A 3dxjB 4qhzD 2zdrA 2j4hB 1c9kC 2x5zA 1e0jB 3e7eA 4m8oA 3d2jA 3hhiA 3fesA 2zxrA 3melC 3h4sA 3au0A 4h16A 3ibsA 4af5A 1txuA 3keoB 3nz2C 1fgsA 3d6aA 4iikA 4f9aC 4gyiA 1tkdA 1lp1B 1mxgA 3uwkA 3vmmA 3lghA 3g2fA 3f1fT 3g1cA 4ifdJ 1lm3B 4cgkB 3zc0B 4q57B 2yjgA 3f1tB 2qg8A 4g9bA 3e54B 1k6dB 2zxuB 3mwcA 3ckgA 3n7xA 3nc3B 3flkC 3cg4A 4h2uB 2izoC 4cw7H 3w15A 1mogA 1tk6C 2wefA 3s70A 2dlcX 1agrA 3zdbA 1hyoB 4bhqA 1slhC 1mauA 9rubA 3e2vA 3ec2A 4gp2A 3twbC 1xedA 3akkA 2b56A 3kgxA 2dqbC 3s5mA 2iiiA 1ii0B 2j7qA 4gdzA 1ozgA 2jk1A 1xu4A 1xz8A 3sb5A 2h5nA 2bifA 4b43A 3sh1C 2g9zA 4biwA 3lijA 3tptB 2pkeA 4i4tF 3crlB 1vsdA 4ayxA 4ofzA 2v0nA 4o2bA 3ai9X 2ceaC 3qbmA 1qs0A 4p0pA 1pt6B 2zy9B 3eqiA 4i0uF 3al5B 2hx0A 2j17B 2vd3A 1ezwA 3c5hA 3fm5C 3tlxA 2xbuB 2qb8A 1g9gA 4j7lA 2ap9A 3vmqA 1ecbC 4dpgB 2d0oA 3crcB 3fpiA 1d3yB 3o61D 3qnmA 4ksiA 3jwgA 3iivB 4a7zA 2yc4C 2obbA 3f2aA 4kgqC 3gybA 4ay2A 3cbgA 1rddA 4f1mA 3sahB 2vedB 3dc7B 4cw7A 1ydfA 1yd1A 2xo6A 3b2sA 4qcr6 4efcA 1vs0A 3ngoA 1dj9A 4ghlA 2gj8D 2ywrA 4gmjB 1f8iB 1j1cB 3k4iB 3mgaB 4ffdA 4qgpA 3vkbB 1ksfX 3sm4B 3i2bA 3cmqA 3dkxC 3fhdA 4cs3A 3oyzA 1rk2B 4lrsA 2i89B 3p2lA 3r7wB 1h65A 3ug7A 4ig8A 4k6eA 2jcbA 4otpA 3riiA 4dwoA 3fcsB 4j00B 2aleA 2a5lB 1nugB 2ww8A 4ty0A 2q9pA 4k0bB 3n4pB 2vpnB 4bgaC 4gt8A 4hutA 1ehiA 4brnA 3hzhA 3gygA 2j0wA 3qtpB 1vmaA 2c9eA 3stpA 4fl2A 3ll6B 1aihC 3k1eB 2bhzA 3sn0A 1ynsA 2fn0B 4a0gB 1f51D 3b5iA 4e4sF 3r09A 3edvB 2ae8B 4k7yA 4ottB 4mndA 1v0dA 2o06A 2vk6A 3cnxC 3lacB 3n1eB 2hcfA

Independent evaluation set

3cmeT 3zvlA 2hx1C 2hv6A 1x8bA 3tiqB 3doeA 2y87A 2c5uB 2xj9A 1rlmD 3qxcA 3mtcA 2c3oA 1ys7B 4g61A 2dgnA 4hubL 1we3A 3tavB 1tt4A 4okzD 2fkzD 3fqxA 3dkcA 4ay7B 3i0oA 4ifzA 2yniA 3lopA 1bwvC 1h74B 4dfdA 3nemB 2hkjA 3f79E 1w78A 2q0yA 3mg8L 4ffoA 4qjlA 4fwiB 2gjkA 1g8hB 3s89C 1vx9P 3v23Q 1qu2A 3cmwA 1z72B 1wpvB 4d09B 1ig5A 4kxwA 1aqfH 2fcoA 4qcr2 3fkqA 3ct7E 4b3tN 3tw6D 3cq3D 3q45A 4hnzJ 3ahcA 4kj53 1m3uI 3qfvB 1m1bA 3oevK 2q5zB 4klnA 1amuA 3jz0B 1svwA 4c7yA 2pk0A 1jd2G 1ovdA 3ey9B 1uutB 2whiE 2i5rB 3h87A 2r8eH 3ejaA 2vzbA 3vnpB 4qcpD 2ihvD 3pdeD 2rcyA 4c0lA 2vbvB 1ne9A 4obbA 3w9tF 3vatA 2hw5D 4as5B 2zrzA 1kd14 1t5jA 1gljY 1cmcA 3ig5A 1urbA 2p06B 4iokB 4f6tB 1q19D 3f1eE 3a7rA 4qcrW 1ryaB 3u4mA 1n0hA 2gx2I 1v14A 3es7B 3fefC 1wpgA 1efkC 4bvrB 1iw7O 3ld0U 1fiuC 4hb4A 3kwsB 4nx8A 3knhO 3eywA 3zvhB 3a4wB 4mzuB 2a69C 3bhtB 3cbtA 4q04A 3h7vA 3nm7A 3hdgA 3ohzF 4nkbA 2vn2A 1dakA 3knhF 1nj1A 4hxgF 2y4mB 4dtfA 4bpwA 4qcz3 2quiA 4qcnH 1q6oB 3c4qB 3lasB 3o5tA 1qhyA 4eo7A 3vayA 1v5fA 2qojZ 3na8C 4qcrN 2yfnA 4qbyI 2g8hA 3ed6A 1cw0A 3c22D 2a9fA 2e67B 2x3jA 1xmqJ 3bwlB 1p5zB 2oiwC 4ltzA 4qcr8 2aerL 4tq4D 1nlqE 2bhdA 4fvuA 1znoB 2pmqB 4dpvZ 3n2nD 4ev0A 3ocvA 4ariA 3k9fC 1h56A 2yevD 2yvmA 4dkwB 2a87A 1sojD 3uqeA 3pdtA 1iruU 1q9sA 5xiaA 1ka1A 1fjgL 1it8A 3d6wA 4ip2C 3koaA 1mowA 4gp7A 3l23A 4cglA 4as2B 3akmD 4pyoB 3au9A 2npiB 2pgoB 3etjB 2o1oA 2icpA 2yi9C 1d2iA 4mpoB 3cmeB 1jylC 4qcr1 1svmC 4nb4F 2px0H 3mf4B 4nvvR 1n32D 2uu7I 2vjlA 2zxcA 4if4D 1b8cA 1gtvA 2zanA 2xhvB 3t5pL 3wgvA 2y4aD 1d9dA 4h0iA 2fbxA 2as8B 2bphB 4nvvG 4gzkA 1ow2B 2b1qA 1miwB 2ad5B 1e1zP 3nvsA 2yxhB 3e48A 3rimC 3v2uD 2hwgA 4bkmA 1fx4A 2ji6B 2g0wB 3fvyA 2j1nB 3mesB 4e6mA 3f1fS 2d2fA 3f61A 3deqD 3r76B 2x7aI 3sduA 2f9rC 2q40A 2amcB 3f85A 2qvrA 2ejwA 1ec9D 4a48B 3v5wA 3obkH 1pg4B 4qczX 2zyrA 2o4gA 2ag0A 1zotA 3v8eF 3s1aA 2dcnB 4q3bD 3qlzB 4cogC 2r42A 3qe6B 3mcoA 1y9aC 4hzdA 2xsqA 4qctO 3fd5B 4nv0B 3o2eA 4kwuA 1rkvA 4hqoA 4amsA 4qczU 3dgtA 3fwsB 3hiyA 2h1cA 2f43B 2dkgA 4kgmA 2yp1C 3sz4A 3en9A 1t3nB 4b3tG 3f1eX 3t5tB 4dzhA 2g25A 3cinA 3h7cX 4miwA 1ytuB 4c3iB 4knhB 2v54B 4be1A 3s9fA 3gnzP 2hvqA 3qy7A 4a8mQ 4g6hA 3dxjD 1q3uA 4f8eB 1r2rD 1sxjD 3k57A 4njiA 1nbmC 3r9xA 1xxxC 2oekA 2fp0A 3wqmA 2aqxA 4q1vA 3g1bB 3kni0 1kxgA 4flxA 2vf7C 4a01B 2yhgA 4lrzD 4dn1A 1musA 2hsjD 2p3nC 3r4cA 2bz0A 1rfqB 3c8vC 3s7zB 4dmzB 3f1eT 4kemB 3i6tB 2jd4B 3cifB 2feaA 2ggeD 2d5fB 1eyzA 3gieB 2aekB 4m83B 3mp3C

1. Mn^2+^

5-fold cross validation set

2ocgA 1htoC 3ze0D 3ldsA 4eweA 2if8A 4h0dA 2cixA 3fxlA 4k28B 4m0vC 8icpA 4ls9B 4nj4A 1kgpC 4ifzA 3llmA 2zadB 3lopA 1gq2N 4ewvA 4ccnB 2hxgC 2o1eA 4oa9A 1uvmA 2b2kB 3kquE 3s23A 1m0dC 3pifC 4fliA 3orkA 4e2qA 1xldA 1txoB 3ol8A 1g0iA 4gikB 3t7mA 3ufxB 3csbA 1de9A 4qkfA 1bxrC 1de6A 4eemA 3hq1B 3ea3B 3ctzA 3w42A 3telA 3ivdA 1gv3B 1i0bA 2bo8E 1h48D 3gg7A 3vylB 3b59D 1s3nB 1b8aB 4edkA 3fcmB 3n3cA 1w2zB 1kflA 1bfrB 2wodB 3m5qA 3nqwA 4ipiA 1oi8A 1wrnB 1fggA 4ewtC 4k1cA 3ckqA 2yb1A 3n9bB 1jqcB 1woiF 1peyB 2uybA 2z86C 4m8dD 4e1dA 2ongB 3idbA 1yd3A 2qjtB 4e7iA 4lomA 1wao3 3bamA 1ystL 3bsoA 1nb7A 1cnzB 1d8hB 4jh9A 1rqqA 4n7tA 2vs8F 2wymA 3ck2A 4ic1D 2p0nA 3dw9A 2enxA 3q23A 4mr0A 4irqC 3ujqA 3frxB 3ilmC 2xi5A 2phnB 1ucgB 4pxbB 2qb5A 2g38B 3pfpA 2ga2A 4pssA 3f05A 4j6oA 3p2uB 2wc9A 2f5cA 3hmkB 2jecD 3f2cA 1n38A 3rvaA 2bffA 1jlkA 2o5aA 1xmsA 1e24A 4gaeA 3v1rA 4e19A 3cm5A 3fyoD 3h0rK 2z7bA 4ogcA 3r3lB 2ztiA 1vj7A 1a76A 1dckB 2x7jB 1xhvA 1fi2A 1dahA 1i5aA 4fbkA 1u3eM 4bzcC 2qgiA 3lzqB 1twfA 4lfiB 2vqrA 1ef2A 2a9iA 2pyjB 2hbkA 1qprF 1fa0B 2v8uB 3dysB 3t9qB 2mnrA 1o99A 2amhA 1ipsB 3r7pA 2palA 2iocB 2b51A 3ojnB 2d7iA 4gpuA 1zp9A 3lf1B 3c5mB 3vnlA 2id0B 1jfzB 3qfkA 1c39B 4bifB 4c8dA 2am3A 2jlbA 2jcdA 1vj2A 3i3qB 3gmeA 1eccB 4migC 1zu0A 1lncE 3v0qA 3bg5A 2pnyA 1up6A 1za0A 3azmE 1g5bB 3g3rB 1jlmA 3n10A 1ro6A 4hcxB 2py7X 2xmoA 4ez1C 1e6aA 2oknB 1p3dA 3repA 4kpyA 4iw3J 2wdeA 3s5aA 1w4aC 1a0dC 2faqB 2vbeA 3rmjB 1zaoA 3fijH 3h8fF 4dm0A 2e6cA 1xmfB 4pcaA 3a4kD 4ln7A 2w5qA 3nqbA 3gbrB 3g0zA 2q0mX 3pzlB 3qszA 1n1pA 3gfzB 3u95B 1cw1A 2yf3D 3d2oA 4i2cA 3qh8A 1sx5A 3ib7A 1o4tB 1ss9A 3psnA 1vsfA 2qvwA 4l78A 2alyB 1i19B 2iieA 4nfwF 1imdB 2wjfA 1qpsA 4bulA 1jqnA 3tc3B 1s4pA 3qb5K 1wsgD 3pyzA 2jebA 3ki9A 2bvlA 2pmlX 4eayC 3thoB 3iduA 4lilA 4ggfV 2zxqA 3rl3A 1ybuA 2glfA 1mqwA 1elsA 3mpbB 1itwD 3zt9A 1y63A 3vesA 4g2cB 4kirA 3mxaA 2r8dA 2dtiB 1bvaA 1rm0A 2ns6A 3w5wA 4d8gB 3sl1A 3ulqA 2p7pF 2j46B 1tc2B 3qebZ 2kfnA 3dx5A 4njjB 3ounB 2pomA 1r8mE 4fixB 4imaA 3r5uB 3ke6A 2indA 1t6bY 3o3hA 3weiA 3ob8D 2chrA 1gldG 2qb0B 2zofB 3mz4B 4mynA 1fsaB 1wljA 2guiA 1f66G 3wqoA 2w42A 2zxpA 2f89F 2a8pB 3godA 3mfuA 4c21B 3acaA 3oumA 2ojwC 2yl8A 3a6uA 3zf8A 2i6qA 4g3hC 2pokA 3dkxA 1ii7A 3zk4B 4dwrB 3tr8B 2hzyB 2yesB 2gvdA 4dz4B 1ystM 3a6kA 3q4qA 1qgqA 2fc0A 2ydtA 3ngfB 1jstC 1zm8A 1j25A 3uagA 1r2mA 3dt7A 4q3vC 3tuxA 3rvrB 2iryA 4cswB 3hqrA 2v8jA 4amqA 4g24A 2wzfA 1qh3B 4mdaA 3jyfA 4phrA 2o14A 3exeA 3nvtA 4qrnA 1xvlA 4a25C 3pztB 2j3mB 4lumB 1bowA 3hb3A 3qxbA 3jzvA 1on8B 2f6kA 4hqnB 1ncyA 1khwB 1frwA 4ilkB 3r0lD 2ygkA 2j0bA 1rzdA 2dfjA 2d0cA 2fv2A 3ov7A 2p73A 4fm1A 1twfB 1igvA 3qfmA 2gvdC 3m0vC 2qb6A 4qsfA 1v7pC 3s6vA 1k4lA 1ut5B 2whxA 1oywA 4ac8A 3hb3B 1jaiA 1nvmA 1qmgA 1u8xX 4lt5A 1z25A 2vicA 3fa3B 2nrzA 4hnoA 1xzwB 2ic3A 4db1B 1v1fA 1mavA 2g8iA 3azeD 4pegC 4efdF 4dqwB Independent evaluation set

2ocgA 1htoC 3ze0D 3ldsA 4eweA 2if8A 4h0dA 2cixA 3fxlA 4k28B 4m0vC 8icpA 4ls9B 4nj4A 1kgpC 4ifzA 3llmA 2zadB 3lopA 1gq2N 4ewvA 4ccnB 2hxgC 2o1eA 4oa9A 1uvmA 2b2kB 3kquE 3s23A 1m0dC 3pifC 4fliA 3orkA 4e2qA 1xldA 1txoB 3ol8A 1g0iA 4gikB 3t7mA 3ufxB 3csbA 1de9A 4qkfA 1bxrC 1de6A 4eemA 3hq1B 3ea3B 3ctzA 3w42A 3telA 3ivdA 1gv3B 1i0bA 2bo8E 1h48D 3gg7A 3vylB 3b59D 1s3nB 1b8aB 4edkA 3fcmB 3n3cA 1w2zB 1kflA 1bfrB 2wodB 3m5qA 3nqwA 4ipiA 1oi8A 1wrnB 1fggA 4ewtC 4k1cA 3ckqA 2yb1A 3n9bB 1jqcB 1woiF 1peyB 2uybA 2z86C 4m8dD 4e1dA 2ongB 3idbA 1yd3A 2qjtB 4e7iA

1. Na^+^

5-fold cross validation set

1m4yB 3zk1T 1jz1P 2cd7A 2bmiB 4p33A 3iwjB 1a5uF 1tw8C 3goxA 1glhA 3bk8A 4eiyA 3c0vB 4p4kF 1svyA 3c32A 2dsnA 2ydgA 4jt4B 2bl2E 1jouF 1rv8D 1o8uE 1ba1A 2wuvA 1e3zA 4ntxA 3ndhA 2olnA 2jlnA 3zuyA 2yxuB 3me4A 4eelA 1ag9B 3pncA 3opkA 3b9bA 3lg1A 1lokA 3paqA 3q3gD 3r0xA 3hhqC 1ksuB 3b6pA 1ejaA 2c9rA 2y5hA 3fd6B 2gezC 3ifvC 4jb3A 1i40A 1b57A 4ekfA 1nhjA 4bvnA 2iwfB 2q6hA 3dh4D 3ntuA 2ymeI 3davB 4p5mA 3nvdA 2r25B 1c9oA 2iajA 2j5wA 4g8tB 2x2eD 3dyqB 1suzA 2hzlB 1tc8A 2fmqA 3syaA 1ka0A 2rhjA 3ondB 4kcaB 1nzaA 2zhjA 3biaX 1mwvB 1lkbA 1bgpA 1h80A 1me7A 3nmbA 4matA 1sk4A 4gfiC 1gzgA 2j9yB

Independent evaluation set

1m4yB 3zk1T 1jz1P 2bmiB 4p33A 3iwjB 1a5uF 1tw8C 3goxA 1glhA 3bk8A 4eiyA 3c32A 2dsnA 4jt4B 2bl2E

1. Zn^2+^

5-fold cross validation set

2bcoA 3rpcD 4jivD 3qzcB 4ie6A 1hi9E 3ebgA 1lr5D 1wppA 1wgzA 3eyvA 3bywC 2vnfC 3it7B 3kdkA 2rjbB 2v2aA 3qsuD 1llmC 1q08A 3fnsA 1dzuP 4fwjA 3v24N 2z8fA 4uq7B 1su1B 4griB 3g7lA 1qu2A 4n7sC 1fbxB 2anpA 4oh1A 1mftB 1tnbJ 3tw6D 3balB 3bofA 1ocrS 2jd8K 3mhsC 3r4xA 3v2dY 3ohcD 4koyA 4kfuA 1i50C 1cz0A 2dq4A 1tjlF 2v86B 4c3iI 3sxkA 1xllA 4lc5A 1ychA 4w6zD 1jmuH 3amjA 3l8eA 2oi0A 4ngnA 4mbsB 2g3fA 4k6mB 3ld0R 2xevB 4gnxC 1dq3A 2c20E 4fvkB 4dglB 4ih3C 3ea1A 2zh0F 4e7hA 4k90A 4dwxA 3jygE 3aygA 2f5qA 2jlpD 1kapP 1z83A 4l99B 3fp5A 3mpvB 2gc3A 1dwfM 3ju2A 3dynA 2zc2B 2hf1A 3bknL 1k0wD 3l7tB 3fidB 1hqaA 3oaxA 3wl3A 1rjqA 1ld3A 1xruA 2vxxC 4ov9A 4je7A 2oh3A 2fgyA 2waeA 1t8hA 2pmsD 3msuB 2bteD 4icsA 1it8A 2psrA 4nj5A 4qf3A 1r44E 1jk0A 1q3kC 4g23A 3fkgA 3m2uA 3esjA 3q7jB 2zxcA 3kjiA 2anuF 4qd14 1e67A 3tgnB 2drpD 1khoA 3un6A 3csqD 1q95I 3didA 2g64A 3ltdA 2i14C 4hi8B 3qu6A 2b5lD 4p10A 2bq8X 2jigA 4h9kA 3b6pC 1oaoC 4ehcA 4dlmA 3ad9D 3bvvA 2olmA 1hz5B 4a48B 2gbxD 2byoA 1qq9A 4o62B 1piwA 3v8eF 3gdfB 2bibA 2einC 4o23A 3ndiA 2g2nD 3lggA 3cewA 1y9aC 3g1pB 2nytB 1e7dB 4dzhA 1gl4A 4mhqA 4p6rA 3r0dA 3ib2A 4on1A 2r2dE 4brrD 4r1mA 3ewdA 4f56B 3rgbK 1mbxA 2o6dA 4kf9A 3nqzB 2q02D 3pihA 2gwnA 2vrsC 2oajA 1yg9A 3l4kA 4g6uA 2cihA 4lkaA 3nnqB 3h3eA 2feaA 4k88A 2zo4A 4afsA 2i0mA 2ek9A 3mfqA 4eg2A 2zneA 3hcjB 1sedC 1zswA 3csvA 4avxA 4ct0B 4gizD 3q05B 3ttsD 1q3bA 2nvvD 1ibqA 2qq4H 2zwiB 3h8fF 2bx2L 3ifeA 2rhsC 2c3aB 1ndzA 3q7aB 3kycB 4mj7A 1h7rA 3cneD 4jnjA 1vpyA 3tkkA 3w0fA 1p6oB 3ovgA 2h1iA 4nu7C 3ls1B 4onxE 1u0bB 4l9mA 4k0dB 3ngjD 3lx3A 4fuoA 3dowA 2r8qA 3g27A 3e2dA 2vwhA 3cghA 3axsA 1zy7A 2cksB 4fw6C 2axrA 1dv6H 1xcrB 4dt2B 4ixnA 1i0dA 4b87A 4gelB 2ux1G 4ijdB 1tafB 3o14B 3oj6B 2osoA 4gdfE 1kfiB 3eiiB 2fawA 3v4jA 3b5qB 3mmdA 2vl6B 3feqJ 1u10C 3hcsB 2pliA 2nvqB 3cg7A 3v235 4oy8A 1m2vB 3rqzC 4bp0A 2e88A 2fz6A 4pziA 3hpsB 2qeeE 1pmiA 1hw7A 1jkeA 2w8sC 4pt5A 4ntlA 3bocA 3tenC 3v93F 1r23A 3u5eo 4a0xA 3b4nA 2g02A 4a47C 1a7wA 4cshD 2wyhA 1gaxA 3eqtB 3af5A 1cprA 4h3sA 2fk6A 3p24A 2yntC 4lczA 2y33A 4l3kA 4levA 4b6dA 3cqkA 2avuF 4gqtA 3zdrA 3b1bB 2j44A 2c2uA 3ea6A 1cg2D 3e80B 1oi0A 1z3iX 3tbgC 1qvnB 3hftA 2xumA 2gu2A 2erpB 3ivtB 3u5cb 4gyfA 1f4tB 4cogA 7icrA 3oruA 3rsnA 1xv2C 3u5em 2h39B 3w95A 2vo9B 3mf2A 2oodA 2xybA 3rmqA 2uz3D 1hfeT 4gfjA 2hzcA 4fytA 2wknE 2eulA 4pxyB 3fvzA 4qvuA 3avsA 1kaeA 1f35A 3a32A 2ox8C 2jksA 3hwpA 2i56C 3kwoA 3swnA 4b29A 2w88B 3e9qB 1jr3A 3lumA 1glcF 1ro5A 1ohtA 4aiaE 3lwuA 1nlxK 3llaA 3lrqD 4obiA 1qe3A 3qndB 3ij6A 2clbM 3wojA 1t2tA 1yo7A 2y43B 3ksvA 3h90A 1vhdA 3b0xA 4nefA 3u5oC 2fsaA 1ekjG 3qbeA 2bnoB 2wgqB 3m85A 3w2wA 1nvtB 2np0A 4f14A 4ad9A 1r61A 4lw9J 2x5cB 2vunB 4m6rB 3r75B 3fm2A 3draB 2yc5A 3zniM 1q7hA 4c2mA 3r3qA 4f7oB 3qlaA 1q2rA 4nyuA 2rb4A 2yi1A 4gwdB 2gyqA 2f9kF 1iujB 2c1dA 4hdtA 3v77D 1p6dA 4dwcA 4f9aB 2e6lA 2p18A 1enrA 4hexB 1cu1B 4hc7A 2h6lA 2f9iB 3vthA 2pigA 2ragD 2hsiB 3adrA 3rpgC 3a1zC 2a5hD 2pptA 1x6mB 1i1iP 3htrA 3g45A 4o2iA 4mthA 3u5gd 1au1B 1cnqA 2ch9A 4msjA 3eyyB 2chuA 3o47A 1rp0B 4knkB 3jxpA 3t01A 4hyfB 1iwlA 4lfyB 4gx0B 2i2xM 2einB 3gn5A 2i0oA 3qnvA 1y7pB 1mr1C 1ybqA 4k2hD 2fvmB 1r43A 3qbuD 1ck7A 2dgeA 3coqB 1io0A 3vqjA 3dzaC 4kjmA 4lmgB 4pysA 2vkrD 1b20A 1pegA 4msxA 4enlA 2ek0A 2vh3B 2yz5B 1ef0A 3rxzC 4q8gB 1ak0A 4fc5C 4guaC 4filD 3f6hB 4lh8A 4nvv6 2exuA 3floD 1mkmB 4kjgA 1hkkA 4i14A 3llxA 2bo0A 3ht2C 2fbhA 4i2xB 1pszA 4bmjC 2hf9A 4ojzA 3ga3A 3sjaB 2vrzA 3fjuB 1onsA 4k4tA 3irbA 4ql5B 4l6hA 2d0wB 2j0eB 1rqgA 1b71A 1qp9A 3e6uA 2ybpB 1zkpD 3e7gC 1u2wB 3cp0A 1xx4A 4dr8A 1ta9B 1gpcA 4a6eA 3luoA 3h0nA 3q87A 3g8rA 3i9fB 3zxoB 2g7zB 2x3yA 3mx2B 1z1nX 4mi5A 1dcqA 1o7dA 4l44A 2zzeA 3iibA 2hpiA 1dceB 2pdoD 1zh1A 1jr3E 3wrgA 2dfyX 3cngC 3eqnB 4e45N 4lu3A 1j6wA 4c3iJ 3hmfA 3t6pA 2iv0A 2hekB 4iloA 1k83I 4nn2A 1snnB 1k2yX 3q03B 3phxA 4a2cB 1hr6F 4c4aA 3ts2B 2zj7A 3sd9A 1oqjA 2xvaB 2dkcA 2d5mA 1vq0A 2az4B 2ev6B 3uedC 1iahB 3e1zA 1n08B 3n3uA 4u5gA 3g8qA 3rf5A 3favC 1lbuA 2yb5F 3isoA 3m9gA 3qfkA 2vjfA 3gucA 3o0fA 3h5aC 2vcgD 2yheA 2xb1C 3ljuX 1ty2A 3lqiC 4axdA 1sw4A 3ct8A 1odhA 3c9fB 4c3bF 1de5A 3bvoA 4iuvA 4c5yA 3czxC 4bwzA 1btgB 2dphB 2pgqB 3m7kA 1ldjB 3n9mA 4l9pB 2dvtA 2v9kA 1irxB 2p50A 3nybB 3luuA 2qpxA 3icjA 3g4hA 4m3oA 3a1bA 4af1A 3vpbE 3mhsA 1oj7D 2j6aA 1u0aD 2p2lC 3q3qA 3p1vA 3ky9B 4lqxB 3orjA 3qgvA 3ramA 2igiA 4cc9B 3lcnB 3ujzA 4czwA 4fajA 2v1zA 2gx8C 2obaC 1q7lA 3pe8A 3r3rA 3mbgC 3di4A 2gpyB 3ce9C 3efoB 4bz5B 3t3wF 2owbA 4g3mD 1qipD 1i6nA 2a5vA 3rcqA 1t0bG 2h1nA 3goxA 2er8D 1qx0A 2nxfA 3sjpA 2b9dA 1a6fA 1shwA 2nlyA 1cjvC 3bpuA 3h1mA 1zzmA 4a3nA 1lfwA 1ekmA 2e18A 4gndC 2j6xD 3fl2A 4lqfA 2wkxA 4o64C 3t9kA 3cx3A 4cthA 4k1tC 2z2yA 3pm6A 3h6tB 3hugJ 4lgrA 2vqxA 3mp2A 4muqA 1xvxA 1qwyA 3c37A 2vqgH 3caoA 4ka8A 3ohiA 2ygtA 2q1zB 2c6nA 4kepA 3e7lC 3dfkA 2o6iA 2rccC 3odcB 1fioA 1j2xA 3m4gF 4hp3C 1ci3M 4qn1A 3lu2B 3poaA 3kdeC 2wjyA 3zbwA 4fo9A 3qahA 3gyyD 2z72A 2fidB 3aiiA 2aa4A 1mh2A 4lx9A 2vhfA 3iabB 3qsvA 3hnaA 2i9wA 3m6iB 1zdeA 2xoyB 1r4vA 2cs7A 2xcmF 1wbqA 2gviA 1dk4B 3kyaA 1gleG 2xqnT 3u3dA 4cj1A 4a7zA 1hp7A 3k6jA 2q7sB 2z94A 3d00A 1noyB 1lhnA 1zfnA 1zkjA 3n55A 3t3oA 3m52A 2owoA 3li2A 2ar3B 2z58B 2zsgA 2x80A 4olsA 4me3A 1udvA 1hxrB 2xy4A 1taqA 2xsnD 4l63A 3khiA 1l0yA 1hk8A 2imsA 1waaF 1p9eB 3ayvD 2ejcA 1c8yA 4hx3C 1x1vB 1q1yA 1c3rA 1zzhB 3qdhA 4p3xA 2ushB 1eh6A 2yv5A 1kq0A 3m7pA 4jqpB 4fglD 3sb5C 4mtuA 1gupA 3a9lB 2y20F 1v0dA 2x7mA 1vq2A 3urzB 3plwA 3ifuA 2c1gA 3qdfA 3l11A 4fukA 3tdpA 4k7dB 4kfvA 1vj0A 3c2sA 1k9zA 4jjjA 1zfpE 1ddzB 2q0yA 3mduA 3mruB 2y3dA 2h6tA 1wdkA 3ly0B 2eg3A 3ztvA 2xs7A 2nx8A 4c7aA 3rysA 1wczA 4jixA 1ud9A 2fqpC 1no5B 2imzA 1yn4A 4bmbA 1xovA 3ux3B 1gr0A 2p9xD 4u3eA 4fgmA 1y7wA 4iqrF 2x4kB 3l7xA 3f3qA 3uk3D 1cw0A 3u5gf 2w3zA 2qw5A 4axlA 2zumA 3wajA 3nw0A 3k9tA 4dduA 1zmeD 4c98A 4gslA 1ue1B 1m55B 2x3bA 3cjpA 4hcgA 4iaoB 2ra6D 2yikA 4lmyB 1bp3B 7mdhB 3mz8B 2xpyA 2w22A 1p91A 1x1cA 4l8hB 3fvyA 3m3bA 4bhwB 1twfA 1uvqB 2di3A 1i3oE 4b56A 4esnA 1u9kB 4ci1B 3w20A 3m4rA 2a2iA 4ljoA 2zktB 1v15B 3ubfA 1v33A 3d2nA 3u5ij 4h9dA 2yx1A 2innA 1uqwB 3gshA 2gwgA 2a0bA 4txdA 3dxjD 3kdsE 4cosA 2yhgA 1jccC 2ze7A 2e26A 3k6iA 4emeC 4aycA 4mo1A 1mzbA 4p36A 1dosB 3s2mA 4e5vA 2pebB 3lkvA 2hbmA 2ooiA 2agzH 3kaoA 3pw3A 2x98A 3pfeA 2f6sB 4mb7A 3o94B 3ebeC 3obcA 2ab4A 1ocyA 3rpdA 1pzwA 3dxsX 3g0zA 2qt3A 1yj0A 3ntyA 2qquA 3epmA 3hxsA 4ixjB 3vhtB 4k2wB 1yixB 4mahA 1nvbB 2qm1B 3tn1A 3ce2A 2pajA 3pfoB 4nqyA 2vh9B 3eerA 2oogD 2d3kB 3u24A 3psqA 1y02A 4ao7A 4dguA 3na7A 1v47A 2y0oA 2cuaB 2xnjA 3tj9A 2djwB 2j13A 2ceyA 4o5oB 3tasB 1rmdA 3o2gA 3nvoA 3t8wA 4hk5D 4egeA 2z45A 4e45E 3lotD 3eefA 1yw4B 3lnnA 3swnQ 2qnwA 3st7A 2qtvA 2d74B 4fmuA 3c5kA 1vshA 4eogA 4ebbA 3ee6A 2vqjA 3ax1A 3cxlA 2uurA 3tc8A 3kflA 1xc3A 4l58A 4bt2A 2zzwA 4ay8A 2uz9A 2au3A 3st1A 1jocB 3umiA 2ac3A 3favB 2zetC 1gudA 1q14A 4hl2A 4e4wB 3alrC 3i3wA 3mvqF 4areA 2oo4A 2rknA 2xqcD 2nsfA 4ewqA 4q7rA 3kj1A 2gb5A 3vusA 2j4xA 2hc9A 1nltA 3b0aE 3kl7A 2aydA 3l1eA 3u5ga 2qswA 4b6zB 4ngeD 1xocA 3i2dA 3mhhE 4kujB 1q9uB 4cn9A 4ewlA 2oikC 4ne7A 3dciA 1nuiA 2y2bB 2hbaA 3bliA 3mcxA 3qz6A 4fxoB 3iehA 4ggjA 3rcmA 2w9nA 2cjsC 3f2bA 4qhiB 2o6pA 1jazA 3nitA 1oahA 4p53A 1u5kA 2y7iA 2ph1A 3kfuF 2ou3B 1vs0B 4d09A 4axvA 1urjB 2cbiB 3ephA 3d1tA 4hccA 1v72A 4mz7B 2vacA 1eteA 3vpbB 3t9oB 1xkiA 4kavA 2f44A 3iuuA 4au7A 3iciA 4jd1B 1uwyA 3sngA 3lecA 2aqsA 3av4A 1zbdB 3htkC 1eucB 2w0mA 3mbjA 2rc6A 2hjnA 4binA 1qwrA 2dxnB 2wvkA 2owaA 3t92A 2vz5A 1q2lA 3m1nB 3aalA 4hvlA 1lbaA 4lj0A 3vpeA 2gu1A 2eh9A 1bp3A 2wc8D 1d9xA 2fygA 4i11A 2pw6A 4hd5A 3ibmB 4ogeA 1czfB 4k25A 3lmcA 2j7jA 3s6lD 4gxwA 3hkoA 2x4hC 1pb0A 2wazX 3bkfA 1x3wA 3fggA 1vddA 2pkpA 1vecA 2ogjA 1eu4A 2w4lA 4i6vB 1pguB 2xb4A 1vk9A 3sp7A 3o9pA 3iu6A 2x5rA 3b2zF 4bs9A 3l0aA 1umyD 2yr2B 4gwmA 4c2mB 4g1pA 2rhkC 1mwqA 4n0lA 4lzuA 3gglA 1jqgA 3u9gA 1odzB 4n4wA 4m6gA 1dy1A 3hphC 3lvzB 2hjgA 4dyuH 4ii1B 2xz0B 1yewE 2bjrA 1cjvA 4bf7A 3m4wA 3l22A 1kl9A 2g54A 2e46A 2apoB 1cvrA 1pwuA 3swfB 2z00A 3l9wB 1x0tA 3vk6A 1btkB 4i2fA 1wkqA 1py0A 1k24A 1p9wA 3chvA 1ryqA 1vliA 1b0nA 3ta7A 4n0nA 3cosD 4aojC 4ac1X 3rzaA 3dcpA 4ct0A 1s1gB 1r5tD 4mhnA 4iuwA 2ixdA 1ck1A 3mwmA 3r2jB 4m8oA 2qhaA 1l7oB 3n71A 2i13A 1pq4B 4nm6A 2riqA 4e6rA 2dh3A 2fpwB 1txlA 1auiA 3fymA 1vykA 3vrhA 2e7yA 4ndhB 4ifdJ 2xigC 1z2lA 4oteB 3giuB 4nl4H 2czrA 2izoC 4b9pA 3idvA 3go9A 1ve0A 3ivbA 3tp2A 4f6nA 3rhgA 1eb6A 4aapA 2v8lA 3lubF 3m1mA 2cyeC 3h50A 3s5mA 2qkdA 2d74A 4c9zA 2c5wB 2hu9A 1lmlA 1rfuF 2eerA 1xagA 4n4rC 1yt3A 1bi0A 2imrA 2iimA 3q4rA 4iumA 4m0cA 3oa4A 1r8qE 3menB 1i7wC 3sp4B 3ihpB 4mt2A 2qa1A 3e38B 4gbmA 1z05A 3bb6C 2xb3A 1u8bA 1keaA 3vuvA 3n12A 2y6eB 3tp9A 3gl6A 1qrlA 1vheA 4ox3A 2p1hA 1t4wA 3zg6A 3mlpA 3ug7A 2glzA 1su3B 2dt8A 4ivvA 4o6sA 1s4iB 4ccgX 2xoeA 2xl9B 3u5ep 3h7tA 1nyrA 2qgsA 3tmiA 1y9qA 2cjbB 4ladB 3gs2A 3o70A 2wbtA 2nvuB 3ii1A 4gy5A 3hruA 2cd9A 2pg3A 4ap4A 2yjpA 3wi9A 4kywA 4cdgA

Independent evaluation set

2bcoA 3rpcD 4jivD 3qzcB 4ie6A 1hi9E 3ebgA 1lr5D 1wppA 1wgzA 3eyvA 3bywC 2vnfC 3it7B 3kdkA 2rjbB 2v2aA 3qsuD 1llmC 1q08A 3fnsA 1dzuP 4fwjA 3v24N 2z8fA 4uq7B 1su1B 4griB 3g7lA 1qu2A 4n7sC 1fbxB 2anpA 4oh1A 1mftB 1tnbJ 3tw6D 3balB 3bofA 1ocrS 2jd8K 3mhsC 3r4xA 3v2dY 3ohcD 4koyA 4kfuA 1i50C 1cz0A 2dq4A 1tjlF 2v86B 4c3iI 3sxkA 1xllA 4lc5A 1ychA 4w6zD 1jmuH 3amjA 3l8eA 2oi0A 4ngnA 4mbsB 2g3fA 4k6mB 3ld0R 2xevB 4gnxC 1dq3A 2c20E 4fvkB 4dglB 4ih3C 3ea1A 2zh0F 4e7hA 4k90A 4dwxA 3jygE 3aygA 2f5qA 2jlpD 1kapP 1z83A 4l99B 3fp5A 3mpvB 2gc3A 1dwfM 3ju2A 3dynA 2zc2B 2hf1A 3bknL 1k0wD 3l7tB 3fidB 1hqaA 3oaxA 3wl3A 1rjqA 1ld3A 1xruA 2vxxC 4ov9A 4je7A 2oh3A 2fgyA 2waeA 1t8hA 2pmsD 3msuB 2bteD 4icsA 1it8A 2psrA 4nj5A 4qf3A 1r44E 1jk0A 1q3kC 4g23A 3fkgA 3m2uA 3esjA 3q7jB 2zxcA 3kjiA 2anuF 4qd14 1e67A 3tgnB 2drpD 1khoA 3un6A 3csqD 1q95I 3didA 2g64A 3ltdA 2i14C 4hi8B 3qu6A 2b5lD 4p10A 2bq8X 2jigA 4h9kA 3b6pC 1oaoC 4ehcA 4dlmA 3ad9D 3bvvA 2olmA 1hz5B 4a48B 2gbxD 2byoA 1qq9A 4o62B 1piwA 3v8eF 3gdfB 2bibA 2einC 4o23A 3ndiA 2g2nD 3lggA 3cewA 1y9aC 3g1pB 2nytB 1e7dB 4dzhA 1gl4A 4mhqA 4p6rA 3r0dA 3ib2A 4on1A 2r2dE 4brrD 4r1mA 3ewdA 4f56B 3rgbK 1mbxA 2o6dA 4kf9A 3nqzB 2q02D 3pihA 2gwnA 2vrsC 2oajA 1yg9A 3l4kA 4g6uA 2cihA 4lkaA 3nnqB 3h3eA 2feaA 4k88A 2zo4A 4afsA 2i0mA 2ek9A 3mfqA 4eg2A 2zneA 3hcjB 1sedC 1zswA 3csvA 4avxA 4ct0B 4gizD 3q05B 3ttsD 1q3bA 2nvvD 1ibqA 2qq4H 2zwiB 3h8fF 2bx2L 3ifeA 2rhsC 2c3aB 1ndzA 3q7aB 3kycB 4mj7A 1h7rA 3cneD 4jnjA 1vpyA 3tkkA 3w0fA 1p6oB 3ovgA 2h1iA 4nu7C 3ls1B 4onxE 1u0bB 4l9mA 4k0dB 3ngjD 3lx3A 4fuoA 3dowA 2r8qA 3g27A 3e2dA 2vwhA 3cghA 3axsA 1zy7A 2cksB 4fw6C 2axrA 1dv6H 1xcrB 4dt2B 4ixnA 1i0dA 4b87A 4gelB 2ux1G 4ijdB 1tafB 3o14B 3oj6B 2osoA 4gdfE 1kfiB 3eiiB 2fawA 3v4jA 3b5qB 3mmdA
