# Supplementary material for: The Identification of Metal Ion Ligand-Binding Residues by Adding the Reclassified Relative Solvent Accessibility
Source: Front Genet. 2020 Mar 19;11:214. doi: 10.3389/fgene.2020.00214 (PMC7096583; doi:10.3389/fgene.2020.00214)
Supplement: Supplementary file 5 [file Table_1.doc]

**The four grouping methods of relative solvent accessibility of metal ion ligands**

| Ligand | SA_2 | SA_4 | SA_P | SA_V |
| --- | --- | --- | --- | --- |
| Zn^2+^ | $\left\{\begin{matrix} P,x\in[0,0.25) \\ Q,x\in[0.25,0.85] \end{matrix}\right.$ | $\left\{\begin{matrix} E,x\in[0,0.2] \\ F,x\in(0.2,0.45) \\ G,x\in(0.45,0.6] \\ H,x\in(0.6,0.85] \end{matrix}\right.$ | $\left\{\begin{matrix} I,x\in[0,0.5) \\ M,x\in[0.5,0.85] \end{matrix}\right.$ | $\left\{\begin{matrix} A,x\in[0,0.25) \\ D,x\in[0.25,0.85] \end{matrix}\right.$ |
| Cu^2+^ | $\left\{\begin{matrix} P,x\in[0,0.25) \\ Q,x\in[0.25,0.85] \end{matrix}\right.$ | $\left\{\begin{matrix} E,x\in[0,0.2] \\ F,x\in(0.2,0.45) \\ G,x\in(0.45,0.6] \\ H,x\in(0.6,0.85] \end{matrix}\right.$ | $\left\{\begin{matrix} I,x\in[0,0.15) \\ K,x\in[0.15,0.3) \\ L,x\in[0.3,0.4) \\ M,x\in[0.4,0.85] \end{matrix}\right.$ | $\left\{\begin{matrix} A,x\in[0,0.15) \\ B,x\in[0.15,0.25) \\ C,x\in[0.25,0.3) \\ D,x\in[0.3,0.85] \end{matrix}\right.$ |
| Fe^2+^ | $\left\{\begin{matrix} P,x\in[0,0.25) \\ Q,x\in[0.25,0.85] \end{matrix}\right.$ | $\left\{\begin{matrix} E,x\in[0,0.2] \\ F,x\in(0.2,0.45) \\ G,x\in(0.45,0.6] \\ H,x\in(0.6,0.85] \end{matrix}\right.$ | $\left\{\begin{matrix} I,x\in[0,0.15) \\ K,x\in[0.15,0.3) \\ L,x\in[0.3,0.4) \\ M,x\in[0.4,0.85] \end{matrix}\right.$ | $\left\{\begin{matrix} A,x\in[0,0.2) \\ D,x\in[0.2,0.85] \end{matrix}\right.$ |
| Fe^3+^ | $\left\{\begin{matrix} P,x\in[0,0.25) \\ Q,x\in[0.25,0.85] \end{matrix}\right.$ | $\left\{\begin{matrix} E,x\in[0,0.2] \\ F,x\in(0.2,0.45) \\ G,x\in(0.45,0.6] \\ H,x\in(0.6,0.85] \end{matrix}\right.$ | $\left\{\begin{matrix} I,x\in[0,0.3) \\ L,x\in[0.3,0.4) \\ M,x\in[0.4,0.85] \end{matrix}\right.$ | $\left\{\begin{matrix} A,x\in[0,0.2) \\ D,x\in[0.2,0.85] \end{matrix}\right.$ |
| Co^2+^ | $\left\{\begin{matrix} P,x\in[0,0.25) \\ Q,x\in[0.25,0.85] \end{matrix}\right.$ | $\left\{\begin{matrix} E,x\in[0,0.2] \\ F,x\in(0.2,0.45) \\ G,x\in(0.45,0.6] \\ H,x\in(0.6,0.85] \end{matrix}\right.$ | $\left\{\begin{matrix} I,x\in[0,0.4) \\ M,x\in[0.4,0.85] \end{matrix}\right.$ | $\left\{\begin{matrix} A,x\in[0,0.2) \\ D,x\in[0.2,0.85] \end{matrix}\right.$ |
| K^+^ | $\left\{\begin{matrix} P,x\in[0,0.25) \\ Q,x\in[0.25,0.85] \end{matrix}\right.$ | $\left\{\begin{matrix} E,x\in[0,0.2] \\ F,x\in(0.2,0.45) \\ G,x\in(0.45,0.6] \\ H,x\in(0.6,0.85] \end{matrix}\right.$ | $\left\{\begin{matrix} I,x\in[0,0.15) \\ K,x\in[0.15,0.4) \\ L,x\in[0.4,0.5) \\ M,x\in[0.5,0.85] \end{matrix}\right.$ | $\left\{\begin{matrix} A,x\in[0,0.15) \\ B,x\in[0.15,0.25) \\ C,x\in[0.25,0.35) \\ D,x\in[0.35,0.85] \end{matrix}\right.$ |
| Na^+^ | $\left\{\begin{matrix} P,x\in[0,0.25) \\ Q,x\in[0.25,0.85] \end{matrix}\right.$ | $\left\{\begin{matrix} E,x\in[0,0.2] \\ F,x\in(0.2,0.45) \\ G,x\in(0.45,0.6] \\ H,x\in(0.6,0.85] \end{matrix}\right.$ | $\left\{\begin{matrix} I,x\in[0,0.3) \\ L,x\in[0.3,0.4) \\ M,x\in[0.4,0.85] \end{matrix}\right.$ | $\left\{\begin{matrix} A,x\in[0,0.4) \\ D,x\in[0.4,0.85] \end{matrix}\right.$ |
| Mn^2+^ | $\left\{\begin{matrix} P,x\in[0,0.25) \\ Q,x\in[0.25,0.85] \end{matrix}\right.$ | $\left\{\begin{matrix} E,x\in[0,0.2] \\ F,x\in(0.2,0.45) \\ G,x\in(0.45,0.6] \\ H,x\in(0.6,0.85] \end{matrix}\right.$ | $\left\{\begin{matrix} I,x\in[0,0.25) \\ L,x\in[0.25,0.4) \\ M,x\in[0.4,0.85] \end{matrix}\right.$ | $\left\{\begin{matrix} A,x\in[0,0.25) \\ D,x\in[0.25,0.85] \end{matrix}\right.$ |
| Mg^2+^ | $\left\{\begin{matrix} P,x\in[0,0.25) \\ Q,x\in[0.25,0.85] \end{matrix}\right.$ | $\left\{\begin{matrix} E,x\in[0,0.2] \\ F,x\in(0.2,0.45) \\ G,x\in(0.45,0.6] \\ H,x\in(0.6,0.85] \end{matrix}\right.$ | $\left\{\begin{matrix} I,x\in[0,0.5) \\ M,x\in[0.5,0.85] \end{matrix}\right.$ | $\left\{\begin{matrix} A,x\in[0,0.25) \\ D,x\in[0.25,0.85] \end{matrix}\right.$ |
| Ca^2+^ | $\left\{\begin{matrix} P,x\in[0,0.25) \\ Q,x\in[0.25,0.85] \end{matrix}\right.$ | $\left\{\begin{matrix} E,x\in[0,0.2] \\ F,x\in(0.2,0.45) \\ G,x\in(0.45,0.6] \\ H,x\in(0.6,0.85] \end{matrix}\right.$ | $\left\{\begin{matrix} I,x\in[0,0.5) \\ M,x\in[0.5,0.85] \end{matrix}\right.$ | $\left\{\begin{matrix} A,x\in[0,0.55) \\ D,x\in[0.55,0.85] \end{matrix}\right.$ |
